# In Situ Study of Hydrogen Permeable Electrodes for Electrolytic Ammonia Synthesis Using Near Ambient Pressure XPS

**DOI:** 10.1021/acscatal.2c03609

**Published:** 2022-10-27

**Authors:** Davide Ripepi, Boaz Izelaar, Dylan D. van Noordenne, Peter Jungbacker, Martin Kolen, Pranav Karanth, Daniel Cruz, Patrick Zeller, Virginia Pérez-Dieste, Ignacio J. Villar-Garcia, Wilson A. Smith, Fokko M. Mulder

**Affiliations:** † Materials for Energy Conversion and Storage (MECS), Chemical Engineering Department, Faculty of Applied Sciences, Delft University of Technology, 2629 HZDelft, The Netherlands; ‡ Department of Process and Energy, Mechanical, Maritime and Materials Engineering, Delft University of Technology, 2628 CBDelft, The Netherlands; § Department of Inorganic Chemistry, Fritz-Haber-Institut der Max-Planck-Gesellschaft, Faradayweg 4-6, 14195Berlin, Germany; ∥ Helmholtz-Zentrum Berlin für Materialien und Energie GmbH, BESSY II, Albert-Einstein-Straße 15, 12489Berlin, Germany; ⊥ ALBA Synchrotron Light Source, Carrer de la Llum 2-26, 08290Cerdanyola del Vallès, Barcelona, Spain; # Department of Chemical and Biological Engineering and Renewable and Sustainable Energy Institute (RASEI), University of Colorado Boulder, Boulder, Colorado80303, United States

**Keywords:** NAP-XPS, in situ, hydrogen permeation, ammonia synthesis, nitrogen, adsorption

## Abstract

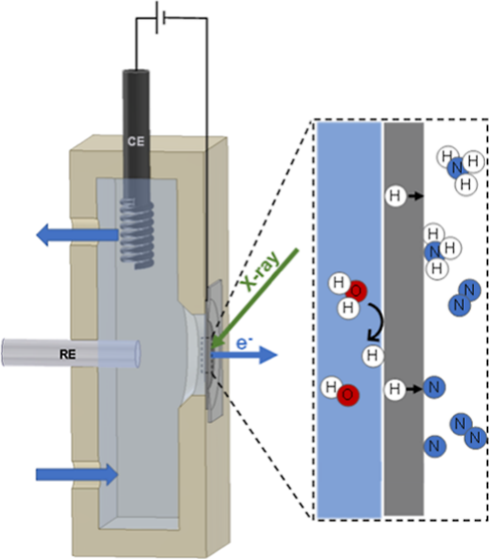

Hydrogen permeable electrodes can be utilized for electrolytic ammonia synthesis from dinitrogen, water, and renewable electricity under ambient conditions, providing a promising route toward sustainable ammonia. The understanding of the interactions of adsorbing N and permeating H at the catalytic interface is a critical step toward the optimization of this NH_3_ synthesis process. In this study, we conducted a unique in situ near ambient pressure X-ray photoelectron spectroscopy experiment to investigate the solid–gas interface of a Ni hydrogen permeable electrode under conditions relevant for ammonia synthesis. Here, we show that the formation of a Ni oxide surface layer blocks the chemisorption of gaseous dinitrogen. However, the Ni 2p and O 1s XPS spectra reveal that electrochemically driven permeating atomic hydrogen effectively reduces the Ni surface at ambient temperature, while H_2_ does not. Nitrogen gas chemisorbs on the generated metallic sites, followed by hydrogenation via permeating H, as adsorbed N and NH_3_ are found on the Ni surface. Our findings suggest that the first hydrogenation step to NH and the NH_3_ desorption might be limiting under the operating conditions. The study was then extended to Fe and Ru surfaces. The formation of surface oxide and nitride species on iron blocks the H permeation and prevents the reaction to advance; while on ruthenium, the stronger Ru–N bond might favor the recombination of permeating hydrogen to H_2_ over the hydrogenation of adsorbed nitrogen. This work provides insightful results to aid the rational design of efficient electrolytic NH_3_ synthesis processes based on but not limited to hydrogen permeable electrodes.

## Introduction

Ammonia (NH_3_) is an essential source of activated nitrogen, required for the global production of N-based fertilizers (more than 180 million tons per year).^1^ In addition, the demand for ammonia is expected to grow because of its potential application as a future energy carrier.^2,3^ The negative environmental impact of the current fossil-based industrial NH_3_ synthesis process,^4^ the Haber–Bosch process, operating at high temperatures and pressures, urges for a more sustainable alternative. The renewable energy-driven electrosynthesis of ammonia from dinitrogen (N_2_) and water under mild conditions may represent an attractive solution to produce fossil-energy-free NH_3_.^2^ Although the electrocatalytic reduction of nitrogen to ammonia has gained large scientific interest,^5^ the hydrogen evolution reaction (HER) strongly dominates over the nitrogen reduction reaction (NRR) in aqueous environments due to the preferential hydrogen activation.^6−9^ The lack of a selective catalyst for nitrogen activation drove the scientific community to explore other approaches to minimize and suppress the competition with HER.^10−14^

**Figure 1:**
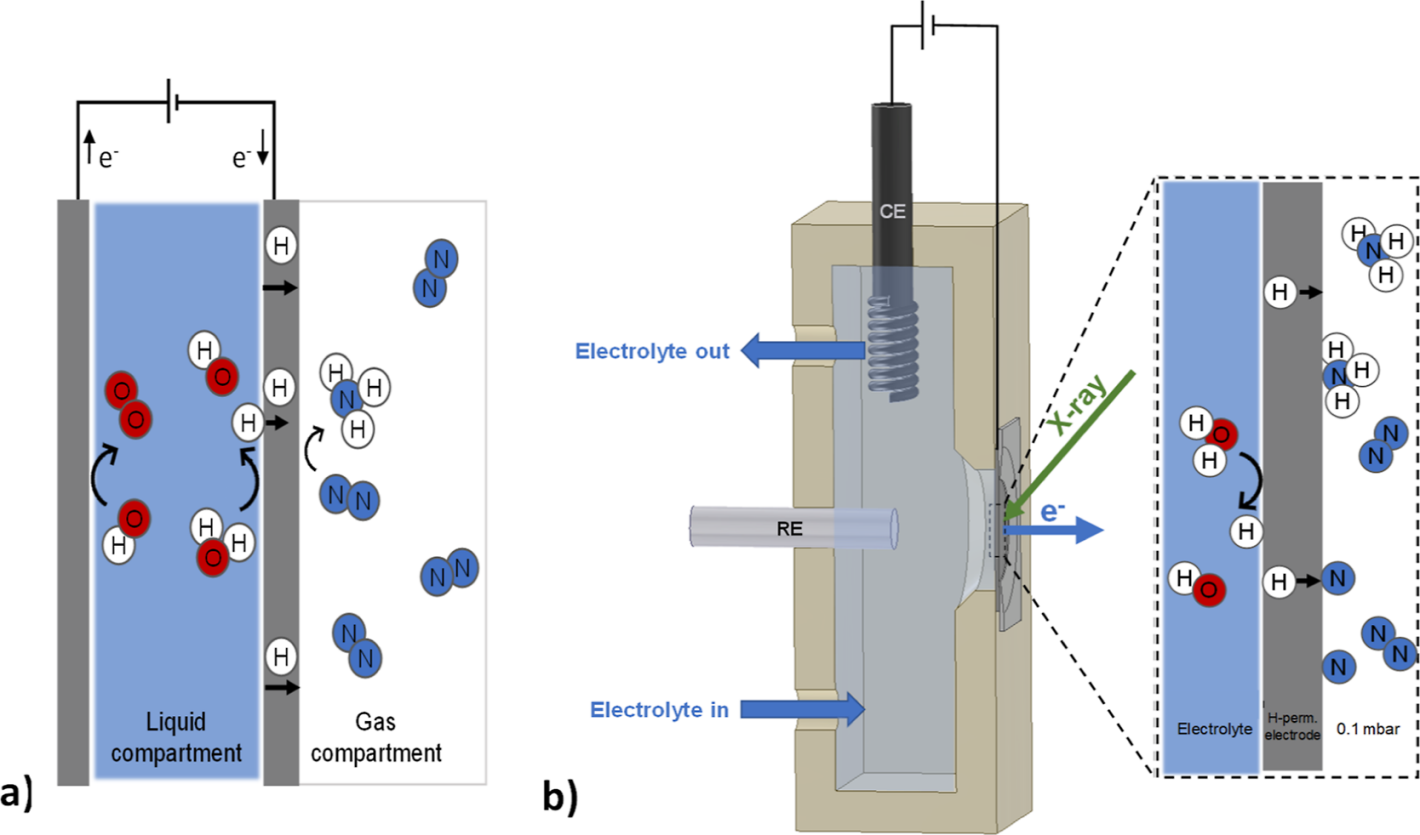
On the left, a schematic representation of the electrolytic cell for ammonia synthesis under investigation, which uses a hydrogen permeable electrode (a). Reproduced from ref (11). Copyright 2021, American Chemical Society. A detailed description of the working principle can be found elsewhere.^11^ On the right, a schematic cross section of the in situ electrochemical flow cell for near ambient pressure X-ray photoelectron spectroscopy used in this study (b). The electrolyte is continuously circulated through the cell. Soft X-ray photoelectron spectroscopy was used to investigate the solid–gas interface (right side of the electrode). The enclosure highlights the main steps involved in electrochemical hydrogen insertion, dinitrogen activation, and nitrogen reduction to ammonia.

In this context, the adoption of a hydrogen permeable electrode proved to be a successful strategy to spatially separate the aqueous electrolyte and the hydrogen reduction side from the N_2_ activation and hydrogenation sites (Figure 1a).^11^ In this way, N_2_ can bond onto the catalytic surface in the absence of other competing adsorbate molecules from the electrolyte. NH_3_ is then formed on the catalyst surface from the hydrogenation of adsorbed N via electrochemically permeating atomic hydrogen under ambient conditions. In this way, when the NH_3_ desorbs, it becomes available directly in the gas phase, without the necessity of further separation from a liquid electrolyte. While the catalytic reaction was demonstrated as a proof of principle with strict isotope labeling control experiments using a Ni hydrogen permeable electrode,^11^ other materials might speed up the rate of the reaction. Iron and ruthenium are among the most promising catalysts according to their reported activity toward nitrogen activation^15−19^ and hydrogen permeability^20,21^ under near ambient conditions. Moreover, in our previous study, we have seen how the presence of surface nitrides, besides protecting the reactive Ni surface against oxidation, may act as a precursor for a low-temperature Mars–van Krevelen (MvK) mechanism.^11^ This two-stage mechanism consists of, first, the hydrogenation of lattice N to yield NH_3_ and a nitrogen vacancy (N^vac^), and second, the filling of the N^vac^ with gaseous dinitrogen.^22^ The use of metal nitrides (MN) as ammonia synthesis catalysts has been proposed and offers several advantages, such as a limited H adsorption.^22−24^ Even though recently there have been some concerns on certain MN used as electrocatalysts for nitrogen reduction reaction (NRR) in aqueous electrolytes,^25^ the catalytic activity of MN was demonstrated with isotope exchange experiments, revealing the occurrence of a Mars–van Krevelen mechanism.^23,26^ Density functional theory (DFT) calculations showed that such surface nitrogen vacancies can activate N_2_ by weakening of the triple bond (N–N bond elongation), lowering the barrier for nitrogen activation.^27−29^ However, the catalytic MvK nitrogen activation typically requires high temperatures to hydrogenate the surface lattice N with reactive H_2_ gas. In contrast, electrochemically inserted and permeating atomic hydrogen reduces surface N to form NH_3_ and N^vac^ at ambient temperature.^11^

Yet, the ammonia production rates achieved until now using H permeable electrodes remain far from commercially relevant applications,^30^ and thus, further mechanistic understanding and technical developments are needed. Consequently, understanding how N_2_ and permeating H interact on the catalytic surface during the reaction can aid the rational design and development of better catalysts. Surface sensitive operando and in situ techniques, such as near ambient pressure X-ray photoelectron spectroscopy (NAP-XPS), can provide such information, as has been shown for several systems recently in the literature,^31−34^ including palladium hydrogen membranes.^35^ XPS commonly operates under ultrahigh vacuum (UHV) conditions due to the short inelastic mean free path length of photoelectrons in a gas atmosphere. Recent technological advances have made it possible to operate XPS under more realistic conditions, bridging the gap between UHV and operating pressure.^36,37^ NAP-XPS can thus measure modifications in the surface electronic structure of the catalyst and the nature of adsorbed molecules in an element-specific manner. Numerous spectroscopic studies on nitrogen adsorption and catalytic ammonia synthesis have been carried out over the years.^15,38−41^ As a result, various adsorbed N species and reaction intermediates have been identified using XPS (Table 1). Remarkably, while extensive studies on the adsorption of N_2_ with XPS are available for Ni and Fe, less work has focused on XPS of nitrogen adsorption on Ru.

**Table 1:** Review of N 1s Assignments of Adsorbed Nitrogen Species on Nickel Surfaces

species	surface	BE (eV)	references
N^ad^	Ni(110)	397.0	(39)
	Ni(100), Ni(110)	397.0	(63)
	Ni(111)	397.8	(59)
	Ni(110)	398.0	(62)
	Ni(poly)	397.8	(60, 61)
	Ni(poly)	397.3	(70)
N_2_^phys^	Ni(111)	405.5^a^	(64)
	Ni(110)	405.3^a^	(39)
	Ni(poly)	405.7^a^	(60)
N_2_^chem^	Ni(111)	401.0^a^	(64, 71)
	Ni(110)	399.4^a^	(39)
	Ni(poly)	400.6^a^	(60, 61)
N_2_O	Ni(poly)	402.0^a^, 406.0^a^	(60, 61)
NO	Ni(111)	399.8	(65)
	Ni(poly)	399.5	(60, 61)
	Ni(poly)	399.4	(70)
NO_2_	Ni(poly)	403.0	(60)
NH_*x*_(*x* = 1,2)	Ni(100), Ni(110)	398.5	(63)
	Ni(111)	399.7	(59)
	Ni(110)	398.4	(62)
NH_3_	Ni(100), Ni(110)	400.5	(63)
	Ni(110)	400.9	(62)

a At temperature ≤80 K.

In this study, synchrotron-based soft X-ray photoelectron spectroscopy was used to investigate the solid–gas interface of nickel, iron, and ruthenium polycrystalline surfaces, and their corresponding nitride phases, under electrochemical hydrogen permeation and a dinitrogen atmosphere. This in situ XPS study shows that the first hydrogenation step to NH and the NH_3_ desorption on Ni might be limiting under the operating conditions. We also provide further evidence of how electrochemically inserted and permeating atomic hydrogen can reduce the surface lattice N on Ni to form NH_3_ and N^vac^ at ambient temperature, while the same process is not observed on Fe and Ru.

## Experimental Section

### Sample Preparation

A total of six types of electrodes were prepared for this study, respectively, Ni, Fe, Ru, and their corresponding surface nitrides. The Ni and Fe electrodes consist of a polycrystalline nickel (12.5 μm) and iron (25 μm) foil, respectively. The Ru electrode consists of a polycrystalline nickel foil (12.5 μm) coated with 20 nm of sputter-deposited ruthenium. All the samples were thoroughly cleaned in an ultrahigh vacuum chamber under Ar/H_2_ plasma for 30 min. The plasma was generated with a radio frequency power supply at 20 W under a constant flow of Ar (35 sccm) and H_2_ (5 sccm). A pressure of 5 × 10^–3^ mbar was held in the reaction chamber by means of a butterfly reducing valve mounted at the inlet of the pumping stage. Surface nitrides were prepared by exposing the cleaned sample surface to an additional plasma nitriding step consisting of an Ar/N_2_ plasma (in 2 to 1 ratio) at 2 × 10^–2^ mbar for 10 min. This results in about a 30 nm thick nickel nitride layer.^11^ The described sample preparation steps were carried out ex situ, prior mounting the electrode in the electrochemical cell. More details on the materials and methods can be found in the Supporting Information. The structural properties of the prepared samples were analyzed with X-ray diffraction (XRD). XRD analysis was performed with a Bruker D8 ADVANCE ECO equipped with a Cu Kα source (1.54060 Å, 40 kV and 25 mA). The Bragg–Brentano measurement geometry was applied with a fixed sample illumination slit of 5.0 mm and a LynxEye XE-T detector. The data were collected at 0.01° step size in the range of 5–110°. The Fe sample was characterized using a Bruker D8 ADVANCE diffractometer equipped with a Co Kα source to avoid the characteristic fluorescence emitted by iron when using a Cu Kα source. The obtained results are included in the Supporting Information (Figures S1–S3).

### Electrochemical Hydrogen Permeation

The electrochemical hydrogen permeation rate of the different electrodes under investigation is measured using the Devanathan–Stachurski (DS) method^42^ (Figure S4). Electrochemical hydrogen insertion is carried out in 0.1 M KOH solution with a galvanostatic cathodic charging current density of 5 mA cm^–2^. The permeating atomic hydrogen is then oxidized at the anodic compartment (hydrogen exit side of the DS cell) at a constant electrode potential of +0.3 V versus SHE in a 0.1 M KOH electrolyte. The measured anodic current is directly proportional to the atomic hydrogen permeation through the specimen. The sample was physically grounded and the multichannel potentiostat set in floating mode.

### Ammonia Quantification

Ammonia quantification is carried out directly in the gas phase with a gas chromatography (GC) method, previously developed in our group. Details of the detection method are available elsewhere.^43,44^ The electrodes under investigation in this study were mounted in a two-compartment polyether ether ketone (PEEK) electrochemical flow cell (Figure 1a). One side of the electrode, in the liquid compartment, is in contact with the aqueous electrolyte (0.1 M KOH), while the other side, in the gas compartment, is in contact with only N_2_ at ambient pressure flowing at 1 mL min^–1^ (the electrode geometrical active area is 2.5 cm^2^). The gas composition of the gas compartment is continuously analyzed via GC. The detection of ammonia was not possible directly at the near ambient pressure XPS setups, mainly because of the small surface area and the large volume of the stainless steel analysis chamber, which is known for high NH_3_ physisorption.^45,46^

### Spectroscopy

The in situ XPS spectra were recorded at two different synchrotron facilities. The in situ electrochemical near ambient pressure X-ray photoelectron spectroscopy experiments were performed at the ISISS beamline at the BESSY II electron storage ring operated by the Helmholtz-Zentrum Berlin für Materialien und Energie using the existing electrochemical flow cell,^47^ as shown in Figures 1b and S5. The electrolyte (0.1 M KOH) was flown in the electrochemical cell via a peristaltic pump with a flow rate of 1 mL min^–1^. The electrochemical experiments were carried out using a Pt counter electrode and a saturated Ag/AgCl reference electrode. The electrochemical insertion and permeation of atomic hydrogen through the metal lattice of the working electrode was achieved with a constant negative polarization of −1.5 V versus SHE (not iR corrected) using a Biologic SP-300 potentiostat. The applied potential matches the operating conditions of our previous work,^11^ resulting in similar charging current density (Figure S6). The operation under potentiostatic conditions was preferred due to better electrochemical stability using the in situ electrochemical cell, compared to galvanostatic operation. The pressure of the XPS analysis chamber reached 10^–7^ mbar prior to starting the experiment. Dosing of the gases in the near ambient pressure XPS chamber was carried out with a set of dedicated mass flow controllers (Bronkhorst) to a pressure of 0.1 mbar. A potential of 90 V was applied to the nozzle of the analyzer to eliminate the gas phase contributions in XPS measurements. The solid Ni hydrogen permeable electrode provides a complete, physical separation between the aqueous electrolyte and the NAP side. Yet, hydrogen atoms can selectively diffuse through the lattice of the Ni electrode, via electrochemical insertion under negative polarization. With this configuration, the reaction between permeating absorbed hydrogen and surface species at the solid–gas interface can be studied in near ambient pressure in the absence of liquid electrolyte.

Near ambient pressure X-ray photoelectron spectroscopy experiments were performed at BL24-CIRCE beamline at the ALBA Synchrotron Light Source, Spain, using a laser-heated sample holder (Figure S5).^37^ The sample was heated from behind using an infrared laser, while the sample temperature was monitored by a K-type thermocouple. The pressure of the XPS analysis chamber prior starting the experiment was about 6 × 10^–8^ mbar and gases were introduced by dedicated mass flow controllers (Brooks). To prevent beam damage effects, each spectrum was recorded on a fresh spot on the surface.

Additional ex situ XPS measurements were collected with a Thermo Scientific K-alpha spectrometer with Al Kα monochromator using a sealed sample holder to avoid air exposure of the samples.

All the spectra in the present paper were recorded with a photoelectron kinetic energy of 200 eV by adjusting the incident excitation energy accordingly, unless stated otherwise. The XPS intensity was normalized by the ring current and photon flux to compensate for slight variations in incident X-ray. More details on both beamlines and data analysis are available in the Supporting Information. All fitting parameters are given in Tables S1–S5.

### Quantitative Model for Overlayer Thickness Calculations

The kinetic energy of the emitted photoelectrons depends on the incident photon energy. Therefore, photoelectrons with different escape depths can be collected by varying the incident photon energy. Variable synchrotron incident photon energy can thus be used to measure the thickness of overlayers in a non-destructive way.^48,49^ The film thickness is derived from the integrated XPS signal intensities and the effective attenuation length (EAL) at different kinetic energies, assuming an uniform overlayer of finite thickness over a semi-infinite substrate, from the following equation
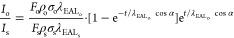
1where *F* is a value that depends on the emitted electron energy and includes various instrumental parameters, ρ is the atomic density, σ is the differential cross section, λ_EAL_ is the effective attenuation length, *t* is the overlayer thickness, α is the photoelectron emission angle measured with respect to the surface normal, and *I* is the XPS intensity from the substrate (s) and overlayer (o). More details on the quantification model are available elsewhere.^49^ The effective attenuation length can be approximated to the inelastic mean free path (IMFP) for kinetic energies above 200 eV.^48^ The values of the IMFP are obtained from the NIST Electron Inelastic-Mean-Free-Path Database (NSRD 71 version 1.2).^50^ The parameters *F* and σ are considered equals for both the substrate and overlayer. The calculated *I*_o_/*I*_s_ values are fitted against the measured ones by stepwise varying the overlayer thickness, until reaching the minimum standard deviation.

## Results and Discussion

### Nickel

**Figure 2:**
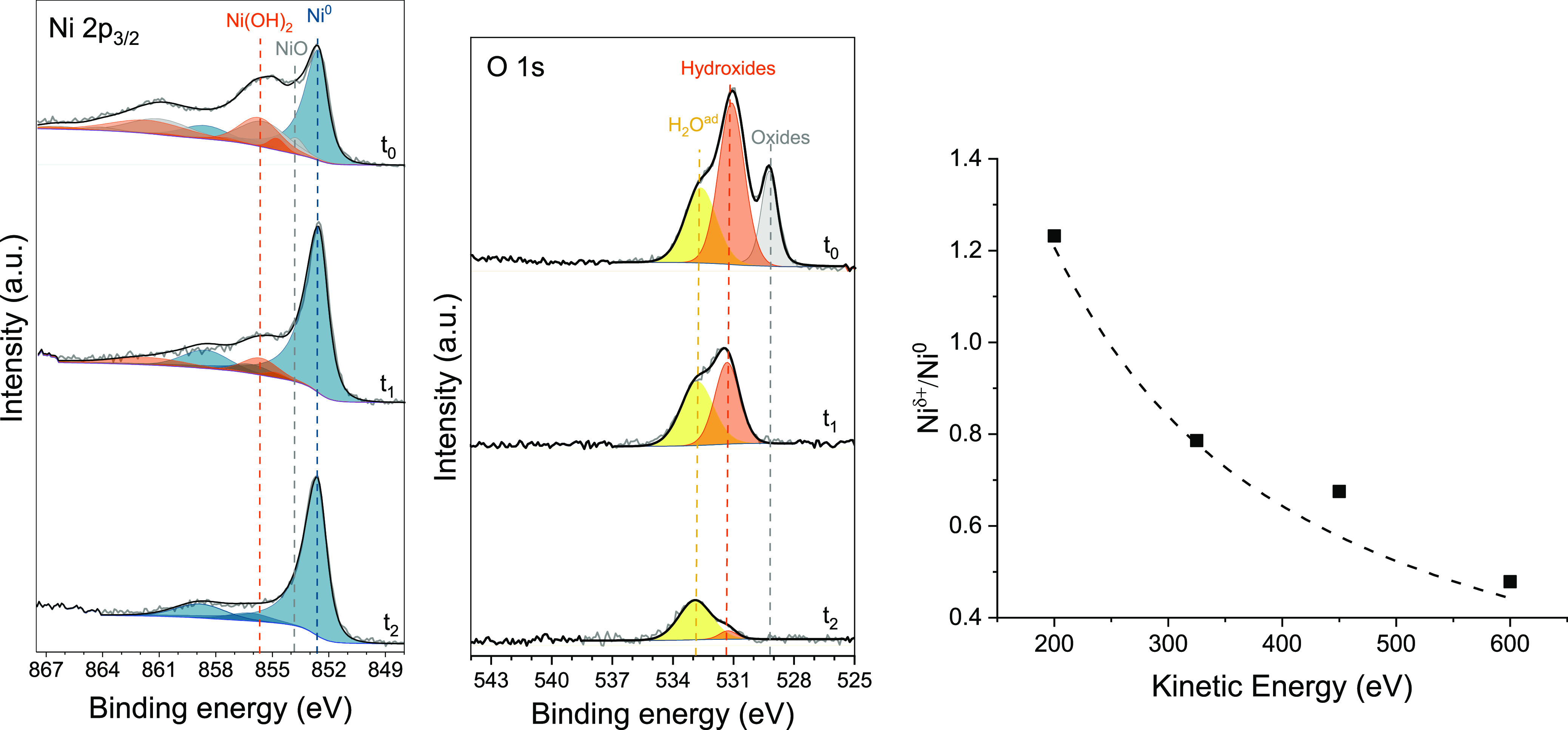
On the left, the Ni 2p_3/2_ and O 1s XPS spectra show, over time (*t*_0_ = 0 min, *t*_1_ = +15 min, and *t*_2_ = +75 min), the in situ reduction of Ni oxide/hydroxide via electrochemically permeating atomic hydrogen at room temperature under vacuum. Fit model using Ni^0^ (blue), metal oxide (gray), metal hydroxide (orange), and adsorbed water (yellow). On the right, the ratio of the Ni 2p_3/2_ XPS peak areas of oxidized and metallic species, at *t*_0_ = 0 min, as a function of photoelectron kinetic energy (between 200 and 600 eV). Measured values (symbols) and calculated fit (dotted line). From this, the initial thickness of the oxidized surface layer is estimated to be 0.75 ± 0.02 nm.

Nitrogen chemisorption occurs on reduced surfaces of various transition metals, even at ambient temperature and pressure.^15^ However, the presence of other reactive species that compete for adsorption sites affects the dinitrogen activation negatively.^6,8,51,52^ Chemisorbed atomic nitrogen has been observed on metallic Ni.^11,15,39,53,54^ In contrast, from our NAP XPS measurements, we found that a NiO layer grown on the polycrystalline Ni does not present any reactivity toward N_2_ adsorption (Figure S7). From these results, it follows, that preserving an oxide-free Ni metal catalytic surface is important to effectively activate N_2_. However, this is a challenge as Ni readily forms surface oxide species even in the presence of traces of oxygen or water due to the highly negative and spontaneous oxide formation energy (about −240 kJ mol^–1^).^55,56^ Hence, it is no surprise that XPS measurements performed on the initially clean metallic Ni foil reveal the formation of a thin mixed oxide/hydroxide surface layer due to the brief exposure to air during sample mounting in the in situ electrochemical setup. The Ni 2p_3/2_ spectrum shows the components corresponding to metallic, oxide, and hydroxide species (Figure 2). The data were fitted according to literature (details are available in Tables S1 and S2).^57^

Consistent with this, the corresponding O 1s spectrum reveals the components characteristic of oxide (529.3 eV), hydroxide (531.1 eV), and adsorbed water (532.9 eV).^57,58^ The thickness of the oxide layer can be estimated using a non-destructive depth profile analysis based on variable synchrotron incident radiation energy.^48,49^ By fitting the experimental data with the simplified model described in eq 1, the thickness of the oxide overlayer is estimated to be approximately 0.75 nm (Figure 2). Details on the quantitative model are available in the Experimental Section. Our in situ observations reveal that, once formed, surface Ni oxides are stable in both vacuum and under gaseous H_2_ (0.1 mbar) at temperatures below 100 °C (Figures S8 and S9). As such, very stringent gas purity would be necessary to maintain a metallic Ni catalytic surface at temperatures close to ambient. However, here, we show that it is possible to apply electrochemical permeating atomic hydrogen to effectively reduce the surface Ni oxides and hydroxides at room temperature (Figure 2). Under electrochemical hydrogen loading, the initial oxidized state of the surface changes. The Ni^0^ contributions become increasingly dominant, ultimately replacing all Ni^+δ^ components. It was observed that Ni oxides are the first species to be reduced, followed by hydroxide components, until a metallic Ni surface is obtained. Oxide decomposition by X-ray or vacuum was ruled out with prolonged measurements under open circuit potential (OCP), which do not show any signs of reduction. This result is expected as the enthalpy of the formation of the surface oxides and hydroxides is rather negative, which indicates that these compounds are relatively stable. Although the reductive action of continuously fed atomic hydrogen maintains a metallic catalytic surface, re-oxidation is observed upon interruption of electrochemical H permeation (Figure S10). This is due to the presence of residual vacuum contaminants, as traces of oxygen and water vapor (the pressure in the analysis chamber prior starting the experiment was about 7 × 10^–7^ mbar).

**Figure 3:**
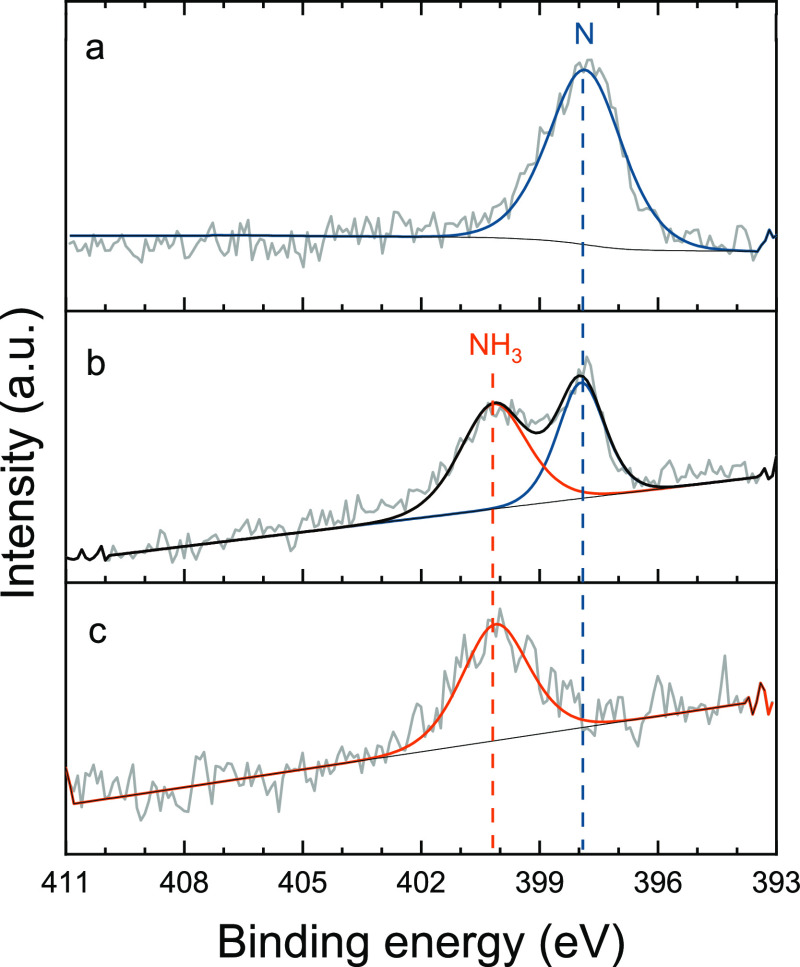
N 1s XPS spectra of clean metallic Ni surface (a) after exposure to N_2_ in OCP, measured ex situ using a sealed XPS sample holder to avoid contact with air (which would hinder the N chemisorption), (b) during the exposure to 0.1 mbar of N_2_ under electrochemical atomic hydrogen permeation, and (c) after subsequent evacuation to UHV under electrochemical atomic hydrogen permeation. The reference spectrum (a) is recorded using a K-alpha spectrometer, while the spectra (b,c) are recorded in situ at the ISISS beamline. The larger width of the peak at 400.3 eV might be due to disorder, which includes different adsorption sites.

Having established how electrochemically permeating atomic hydrogen can in situ reduce the Ni surface under room-temperature conditions, we now turn our attention to the interaction of this reduced surface with dinitrogen gas. Figure 3a shows that a clean metallic Ni surface exposed to N_2_ results in a dominant N 1s peak centered at 397.8 eV corresponding to chemisorbed atomic nitrogen.^41,59−62^ When also electrochemical hydrogen permeation is active, an additional component at higher binding energy (BE, 400.3 eV), assigned to adsorbed NH_3_,^41,59,63^ appears in the N 1s spectrum (Figure 3b). Other nitrogen species on Ni surfaces have been detected at comparable BE, as reported in Table 1. However, molecular dinitrogen on Ni has only been observed at temperatures ≤80 K.^39,60,64^ While NO species can also be excluded, as they have been observed at appreciable lower BE (399.8–399.4 eV) and because of the absence of the respective O 1s component at about 530.3 eV^60,61,65^ (Figure S11). The assignment of this peak to adsorbed NH_3_ is also confirmed from gaseous ammonia detected from a sample with higher N coverage, where a peak at 400.3 eV is also observed upon electrochemical H permeation (vide infra).

Ammonia is thus formed on the Ni surface as result of hydrogenation of chemisorbed nitrogen. However, in this case, on a flat Ni surface with limited N coverage, the ammonia production rate is estimated to be lower than 10^–13^ mol cm^–2^ s^–1^ (corresponding to the instrument detection limit), as no gaseous ammonia was detected with in-line GC in a separate experiment. Reaction intermediates NH and NH_2_, reported on Ni at around 398.5 eV,^59,63^ cannot be discriminated in the N 1s spectra; this indicates that the hydrogenation rate of NH to NH_2_ and NH_3_ is much faster than the hydrogenation of N and the desorption of NH_3_. The persistent presence of atomic nitrogen species at 397.8 eV on the catalyst surface exposed to N_2_ and constant H-permeation (Figure 3b), possibly indicates that atomic N is constantly replenished by newly adsorbed and dissociated dinitrogen. The subsequent evacuation of the N_2_ present in the analysis chamber in OCP (i.e., vacuum conditions without H-permeation) does not alter the N 1s spectrum (Figure S12), indicating that both the N and NH_3_ are stably bound on the surface. However, in the absence of gaseous N_2_ to replenish the reacting adsorbed nitrogen (i.e., under vacuum), the N 1s peak at 397.8 eV progressively disappears when electrochemical hydrogen permeation is restored; leaving NH_3_ as the only N species on the surface (Figure 3c). From these observations, it becomes evident how the presence of stable intermediates and adsorbed reaction products, such as adsorbed NH_3_, limits the availability of the active sites for high rate catalysis. This indicates that NH_3_ desorption and the first hydrogenation step to NH might be limiting factors for the studied process at room temperature. The ammonia adsorption energy on Ni is reported to be about 80 kJ mol^–1^^66,67^ and it desorbs at around 100 °C.^68,69^ Therefore, a slight increase in the operating temperature is expected to significantly improve the performance of the reaction, which will be part of future work.

Following the above rationale, the observed overall reaction on Ni^0^ might still be kinetically slow due to the large activation barrier for the first nitrogen hydrogenation step and NH_3_ desorption. Remarkably, the N_2_ adsorption on Ni^0^ occurs spontaneously until a certain coverage is reached. To overcome these barriers, we extended our in situ study to a modified Ni electrode with a nitrided surface layer, which acts as precursor for a low-temperature MvK mechanism. Here, the rationale is to have a higher density of surface N to facilitate the apparently difficult first hydrogenation step, while the permeating atomic hydrogen in situ generates NH_3_ and a large number of highly active surface N^vac^, accelerating the activation of gaseous nitrogen compared to a metallic Ni site, as described in the following section.

### Surface Nickel Nitride

Nickel nitrides are characterized by low nitrogen vacancy formation energy and relatively low barriers for NH_3_ synthesis,^27,72,73^ making this material a good candidate for the process under investigation. We first verified the stability of the surface nickel nitride under investigation with in situ NAP XPS measurements under a H_2_ atmosphere (0.1 mbar) at room temperature (Figure S13). The stability of the nitrided Ni electrode was also tested at higher temperatures, up to 150 °C, under 1 bar hydrogen atmosphere in a sealed PEEK cell while monitoring the gas composition with a GC. No detectable amounts of ammonia were found within the instrument detection limit (of the order of 10^–13^ mol cm^–2^ s^–1^), suggesting that the nitride surface is also stable under these conditions. These results are consistent with the previously reported inability of a nitrided Ni surface to dissociate H_2_^11^ and other stability reports.^74,75^

**Figure 4:**
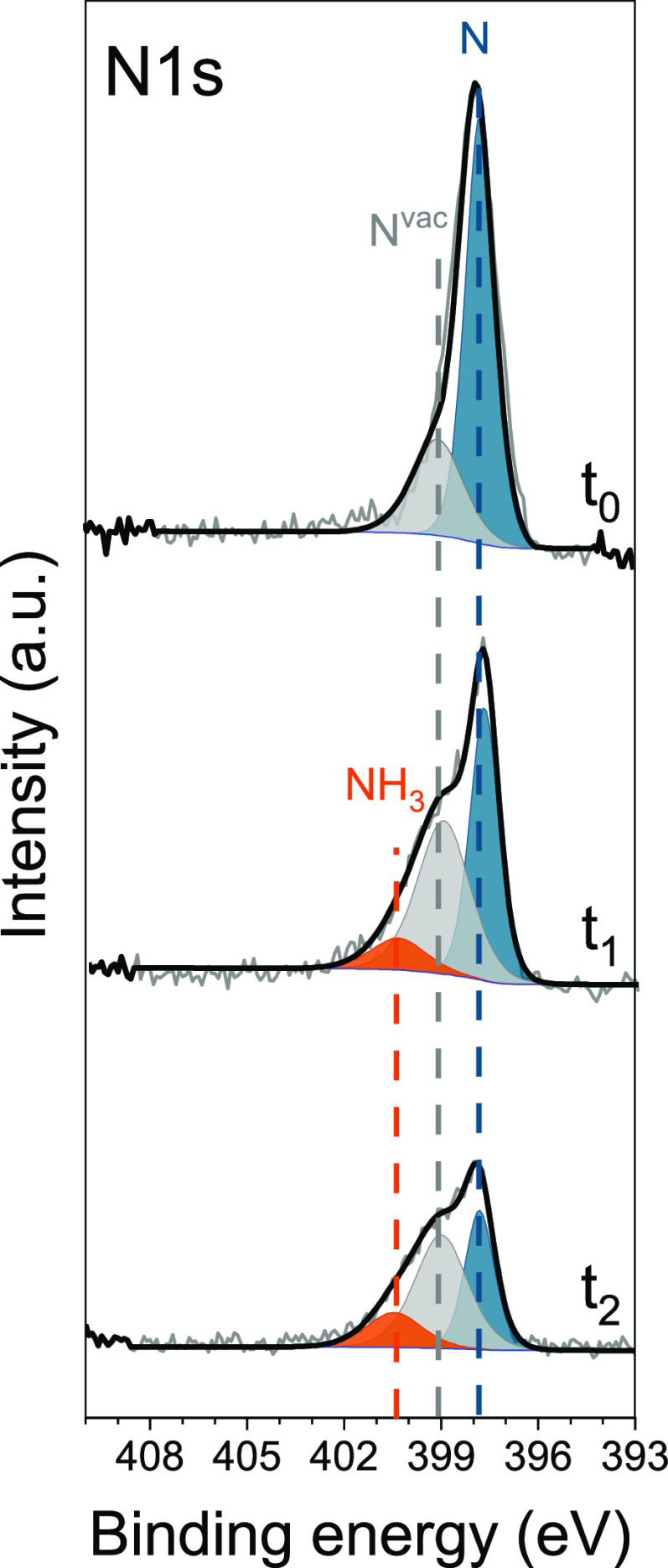
N 1s XPS spectra of the pristine nitrided Ni electrode (*t*_0_ = 0 min) and during electrochemical insertion of hydrogen under vacuum conditions (*t*_1_ = +15 min and *t*_2_ = +90 min). The three components are color coded and corresponding to surface and subsurface atomic N (blue) to N in the proximity of N^vac^ (gray) and to NH_3_ (orange).

On the other hand, the surface lattice nitrogen (dominant component at 397.8 eV in the N 1s spectrum) reacts with the electrochemically inserted hydrogen atoms to form NH_3_, leading to the appearance of a peak at 400.3 eV in the N 1s XPS spectrum (Figure 4), as observed earlier for gaseous N_2_ adsorbed and activated on a clean Ni. However, in this case, the production rate of gaseous ammonia is high enough and is confirmed with GC detection in a separate electrochemical experiment (Figure S14). The presence of stable NH_3_, which remains on the metal surface even after the interruption of hydrogen permeation, indicates that also in this case, product desorption is limiting. However, the high density of adsorbed surface N makes the limiting NH formation step to occur more often and therefore NH_3_ can be produced at a faster rate than with a lower N coverage. In addition, during the hydrogenation of the surface nickel nitride layer, a distinctive new contribution at 399.1 eV appears. This peak is tentatively assigned to N atoms in the proximity of the formed N vacancies on the Ni surface, which results in a shift toward higher binding energy due to the redistribution of electrons left after the hydrogenation of lattice N to NH_3_.^76−80^ The peak appears slightly broader, which might indicate the contribution from N in the proximity of a different number of vacancies. Even though it is difficult to determine the exact nature of this component, we cannot entirely exclude the contribution from some NH_*x*_ species (with *x* = 1, 2), which have been reported on Ni in a similar energy range (Table 1).^62,63^ Moreover, a peak with limited intensity at 399.1 eV is noticeable in the pristine sample (Figure 4), indicating that few N^vac^ might be initially present on the subsurface of the nitrided Ni as result of the plasma nitridation process. Thus, with in situ XPS, it appears possible to directly observe the room-temperature formation of NH_3_ and N^vac^ from reduction of nitrogen adsorbed on Ni via electrochemical atomic hydrogen permeation, while the activation of N_2_ was proven earlier with ^15^N_2_ isotope labeling experiments.^11^

### Iron

Iron has been extensively studied for nitrogen chemistry over the last century, and metallic Fe is well known to activate dinitrogen even under ambient conditions, but still in competition with oxygen and limited by oxidized species.^52^ It is therefore interesting to study a polycrystalline Fe surface under electrochemical hydrogen permeation and a N_2_ atmosphere. The initial oxide layer formed on the Fe foil after a short exposure to air or traces of oxygen represents one of the major limitations in using Fe as nitrogen activation catalyst in a hydrogen permeable electrode system. This layer drastically blocks the atomic hydrogen permeation (Figure S15), preventing the reduction of surface species (i.e., oxides and adsorbed nitrogen) by permeating H. In our case, we measured a reduction in H permeation flux of more than 100-fold (Figure S4). As a consequence, in situ XPS measurements carried out during electrochemical hydrogen loading do not show any significant reduction of the oxide overlayer or the formation of metallic Fe (Figure S16). Only a slight shift toward lower binding energy is noticeable from the Fe 2p spectra, and it can be associated to a limited partial reduction of some Fe^+3^ species to Fe^+2^. Moreover, the highly negative iron oxide formation energy might represent an additional barrier for the oxide removal by permeating atomic hydrogen, compared to nickel oxides.^56^ The higher stability of iron oxide species is also confirmed by our in situ XPS experiments, where temperatures higher than 250 °C under a hydrogen atmosphere (1 mbar) are required to fully reduce the Fe surface (Figure S17). The presence of only a minor metallic Fe^0^ component after cooling of the thermally reduced iron surface under H_2_ indicates that the reoxidation of iron reaches a thicker layer, compared to the oxygen exposed Ni which still shows a strong Ni^0^ contribution (Figure S8). Therefore, contrary to what observed with Ni, the electrochemical insertion of atomic H did not successfully reduce the surface of the iron electrode.

**Figure 5:**
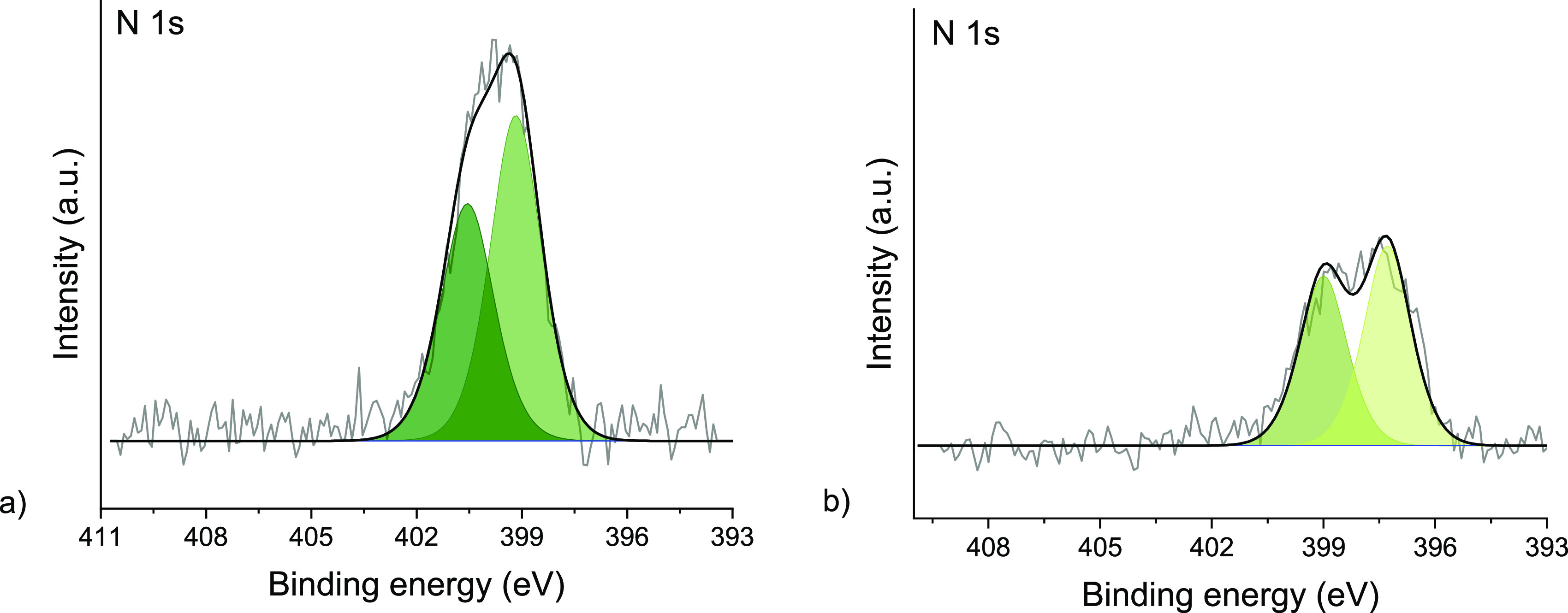
(a) N 1s XPS spectrum of the oxidized polycrystalline Fe under a 0.1 mbar N_2_ atmosphere in open circuit (i.e., no H loading). The N 1s shows two adsorbed nitrogen species at 400.6 and 399.1 eV. (b) N 1s XPS spectrum of the polycrystalline Ru surface under a 0.1 mbar N_2_ atmosphere in open circuit (i.e., no H loading). The N 1s shows two components at 399.0 and 397.3 eV. Photoelectron kinetic energy was 300 eV.

Remarkably, although no metallic Fe^0^ could be observed, when the oxidized iron surface is exposed to N_2_ (0.1 mbar) in OCP, the N 1s XPS spectrum reveals the formation of two nitrogen species (Figure 5a). Adsorbed molecular nitrogen on Fe surfaces has been observed only at extremely low temperatures.^16^ Moreover, as no H_2(g)_ nor permeating atomic H are available (only unavoidable traces of water are present), we do not expect the formation of hydrogenated forms of N. Recently, Degaga et al. reported analogous N 1s peaks on a Fe_3_O_4_(001) surface exposed to N_2_.^38^ Based on their observations and theoretical calculations, these N 1s contributions detected on the Fe oxide surface were assigned to N–O (400.6 eV) and N–Fe^3+^ (399.1 eV) bonds.^38^ This reveals that nitrogen activation is also possible on the oxidized Fe surface. However, upon electrochemical hydrogen insertion, we did not observe any hydrogenation of the adsorbed N species, possibly due to the H blockage by the iron oxide layer, which strongly limits the access of reactive atomic hydrogen to the surface.

A nitride surface layer, generated via plasma nitridation, on a reduced Fe surface can act both as a protective layer against oxidation^81^ and as precursor for a MvK N_2_ activation mechanism. However, the formation of a thin iron nitride surface layer also blocks the atomic hydrogen permeation (Figure S4). As such, no hydrogenation of surface iron nitride is observed during in situ XPS due to the insufficient H flux or too stable Fe–N bond.

### Ruthenium

Both the materials investigated so far (Ni and Fe) are prone to oxidation when exposed to air or water, which blocks N active sites and obstructs the permeation of atomic hydrogen. For this reason, it is interesting to extend our study to more noble metals, such as ruthenium, which is a well-established ammonia synthesis catalyst.^82^ The electrochemical insertion of H through the Ru (and Ru nitride)-coated Ni electrode results in a moderate H permeation flux in comparison to the uncoated Ni (Figure S4).

As expected, the Ru 3p and 3d spectra reveal a metallic surface and the absence of contributions from higher oxidation states (Figure S18). During the exposure to N_2_ (0.1 mbar in OCP), the N 1s spectrum shows two contributions, indicating the presence of dissociated N adsorbed on the polycrystalline Ru surface (397.3 eV) and a second N species at 399.0 eV (Figure 5b). The latter appears at a binding energy close to adsorbed N_2_, whose cleavage is established to be the limiting step for N_2_ activation on Ru catalytic surfaces.^82−85^ However, the acquired data did not provide an unambiguous identification of the nature of this species. Yet, the N 1s spectrum remains nearly unchanged upon electrochemical hydrogen permeation, showing no signs of other N species on the Ru surface in the course of the experiment. Similarly, no substantial variations in the N 1s spectrum are observed for the nitrided ruthenium electrode under electrochemical H loading, although the H-permeation is not blocked by the presence of a surface nitride layer (Figure S4). In both cases, no NH_3_ was detected with gas chromatography in separate electrochemical experiments. Thus, the formation of H_2_ from surface recombination of permeating H prevails over the hydrogenation of the adsorbed N. The stronger Ru–N bond, compared to Ni,^86^ might be too stable to be hydrogenated by H-permeation under these conditions; aggravating the barrier to the first hydrogenation. In fact, Ni has the least negative adsorption energy of nitrogen, among the tested materials, which indicates that the N–M bond follows the trend: Ni < Ru < Fe.^87^ This may be the underlying reason why only Ni shows appreciable NH_3_ formation in this study, next to the more persistent presence of iron oxide. We thus envision that a rational design of catalysts with optimal nitrogen-surface interactions is needed to deploy the full potential of H permeable electrodes for electrolytic ammonia synthesis under near ambient conditions.

## Conclusions

In this study, we used in situ near ambient pressure XPS to investigate the solid–gas interphase of a polycrystalline Ni electrode under electrochemical H permeation and a N_2_ atmosphere for NH_3_ synthesis. The availability of surface Ni^0^ sites is a primary requirement for the chemisorption of gaseous N_2_, as we verified that a fully oxidized Ni surface does not present any reactivity toward dinitrogen adsorption. We showed how electrochemically inserted and permeating atomic hydrogen can reduce surface Ni oxide and hydroxide species, under conditions at which gaseous H_2_ alone does not. Chemisorbed nitrogen is then detected on the metallic Ni surface under a N_2_ atmosphere, followed by the formation of NH_3_ from the reaction with permeating H. The presence of stable adsorbed NH_3_ on the Ni surface, indicates that product desorption might be limiting at room temperature, thus reducing the availability of active sites. The first hydrogenation step to NH may also be limiting, as higher production of NH_3_ is observed when more N is present on the surface. When a thin nitride layer is present, the electrochemically permeating atomic hydrogen reduces the surface lattice nitrogen to form NH_3_ and N^vac^ at ambient temperature, as observed on the surface of the electrode with in situ XPS. These defective sites (i.e., N^vac^) promote the activation of dinitrogen compared to the slower N_2_ activation on Ni. Importantly, the hydrogenation of surface lattice nitrogen was not observed with H_2_ at temperatures up to 150 °C, emphasizing the benefit of electrochemically permeating atomic H.

We extended the study of hydrogen permeable electrodes for NH_3_ synthesis to Fe and Ru catalytic surfaces. The presence of either Fe oxides or nitrides severely inhibits permeating H from accessing the catalytic surface, hindering the advancement of the reaction. Dinitrogen activation is observed on the polycrystalline Ru surface. Still, no evidence of hydrogenation of the adsorbed nitrogen species upon H permeation is found under near ambient conditions. Based on these results, it emerges that H permeability and M–N bond strength are two key parameters to be taken in consideration in the design of an optimal N activation catalyst using H permeable electrodes.

In conclusion, our findings demonstrate the dual benefit of H permeable nickel electrodes, which in situ generate active sites by oxide and nitride reduction at the catalytic solid–gas interface, while providing controllable flux of H atoms available for hydrogenation of adsorbed N. Moreover, we show how in situ NAP-XPS can be applied to investigate processes based on hydrogen permeable electrodes, providing invaluable insights needed for their efficient design.

## Supporting Information Available

Details on sample preparation and characterization, additional XPS characterization, electrochemical hydrogen permeation measurements, gas chromatography for ammonia detection, and XPS fitting parameters (PDF)
