# Supplementary material for: In Situ Study of Hydrogen Permeable Electrodes for Electrolytic Ammonia Synthesis Using Near Ambient Pressure XPS
Source: ACS Catal. 2022 Oct 27;12(21):13781–91. doi: 10.1021/acscatal.2c03609 (PMC9638989; doi:10.1021/acscatal.2c03609)
Supplement: Supplementary file 1 — cs2c03609_si_001.pdf [file cs2c03609_si_001.pdf]

Supporting Information for

In Situ Study of Hydrogen Permeable Electrodes for Electrolytic Ammonia Synthesis using Near Ambient Pressure XPS

Davide Ripepi,[a] Boaz Izelaar,[b] Dylan D. van Noordenne,[a] Peter Jungbacker,[a] Martin Kolen,[a] Pranav Karanth,[a] Daniel Cruz,[c] Patrick Zeller,[c, d] Virginia Pérez-Dieste,[e] Ignacio J. Villar-Garcia,[e] Wilson A. Smith,[a, f] and Fokko M. Mulder [a,*]

[a] Materials for Energy Conversion and Storage (MECS), chemical engineering department, faculty of applied sciences, Delft University of Technology, 2629 HZ Delft, The Netherlands.

[b] Department of Process and Energy, Mechanical, Maritime and Materials Engineering, Delft University of Technology, 2628 CB Delft, The Netherlands.

[c] Fritz-Haber-Institut der Max-Planck-Gesellschaft, Department of Inorganic Chemistry, Faradayweg 4-6, 14195 Berlin, Germany

[d] Helmholtz-Zentrum Berlin für Materialien und Energie GmbH, BESSY II, Albert-Einstein-Straße 15, 12489 Berlin, Germany

[e] ALBA Synchrotron Light Source, Carrer de la Llum 2-26, 08290 Cerdanyola del Vallès, Barcelona, Spain.

[f] Department of Chemical and Biological Engineering and Renewable and Sustainable Energy Institute (RASEI), University of Colorado Boulder, Boulder, Colorado 80303, United States

[*] Email: F.M.Mulder@tudelft.nl

Materials and methods

Potassium hydroxide pellets (Sigma-Aldrich, 99.99% trace metals basis) and Milli-Q water were used for the preparation of the electrolyte solution (0.1 M). The polycrystalline nickel foil with a thickness of 12.5 µm and 99.9 % purity was purchased from Goodfellow. The polycrystalline iron foil with a thickness of 25.0 µm and 99.5 % purity was purchased from Advent Research Materials Ltd. All the samples where thoroughly cleaned in an ultra-high vacuum chamber under Ar/H_2 plasma for 30 min. The Ru thin film was deposited on the cleaned polycrystalline Ni foil by reactive magnetron sputtering (AJA International Inc.) at 100 W DC power in Ar (6N) atmosphere. Ruthenium sputtering target was purchased from Mateck GmbH (purity 99.9 %). Prior to the deposition the pressure of the ultra high vacuum chamber was about 10^{-8} mbar. During the deposition a constant Ar flow of 35 sccm was used and the pressure held to $3 \cdot 10^{-3}$ mbar by mean of a butterfly reducing valve mounted at the inlet of the pumping stage. The deposition time was adjusted to obtain a film thickness of 20 nm, as determined by surface profilometry (with a relative uncertainty of about 10 %). Analogously to the Ru sputtering deposition, also the protective 20 nm Pd layer for the hydrogen permeation experiments in the Devanathan-Stachurski setup was prepared by reactive magnetron sputtering, using a 2-inch metal target (MaTeck, 99.9 % purity) at 100 W DC power in an Ar atmosphere.

X-ray photoelectron spectroscopy

The ISISS beamline of BESSY II employs bending magnet (D41) and a plane grating monochromator (PGM), tuneable photon energy 80-2000 eV, detection of photoelectrons normal to surface, 0.1 mm x 0.08 mm beam spot size and $6 \cdot 10^{10}$ photons/s/0.1 A at a 0.111 mm exit slit. The NAP-XPS end-station is equipped with a SPECS PHOIBOS 150 NAP hemispherical XPS analyser. The spectra were collected using 10 eV pass energy (20 eV for survey scan) and 0.1 eV step, with an experimental resolution of about 0.55 eV. All the gases (N_2, O_2, H_2) were supplied by Westfalen AG and had a purity of 99.9999%. The *in situ* electrochemical cell is made of polyether ether ketone (PEEK). The sample size for the H permeable electrode was 10 x 10 mm, with a geometrical active area of about 50 mm^2, defined as the inner area of the O-ring that contains the electrolyte.

The BL24-CIRCE beamline at the ALBA Synchrotron Light Source uses tuneable photon energy 90-2000 eV, beam incident angle 80 ° from normal to surface, beam spot size 0.1 mm horizontally and 0.03 mm vertically. The NAP-XPS end-station is equipped with a SPECS PHOIBOS 150 NAP hemispherical XPS analyser. More details on the BL24-CIRCE are also available elsewhere.[2] The spectra were collected using 10 eV pass energy (20 eV for survey scan) and 0.1 eV step, with an experimental resolution of 0.29 eV. All the gases (N_2, H_2 and O_2) used had 6.0 purity grade.

Data analysis was performed using CasaXPS software. For each core shell, a valence band spectrum was recorded at the respective photon energy for a proper energy calibration.[3] The absolute binding energy values were allowed to vary by ±0.1 eV. Because of spin-orbit coupling, Ni 2p, Fe 2p and Ru 3p spectra are split in two parts ($p_{3/2}$ and $p_{1/2}$), with a defined intensity ratio. In this case, only one part of the spectrum ($p_{3/2}$) is used for fitting of the components, as common practice.[4]

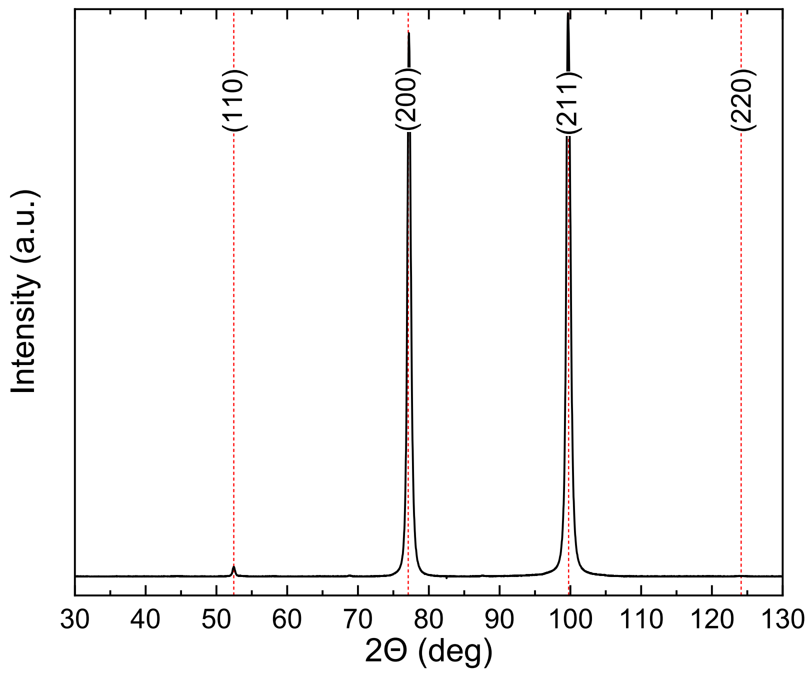

Figure S1 – XRD pattern of polycrystalline Fe foil. The reference diffraction pattern of the α-Fe metal cubic phase (PDF 04-014-0360), is shown as red dotted vertical lines (Co K_α source).

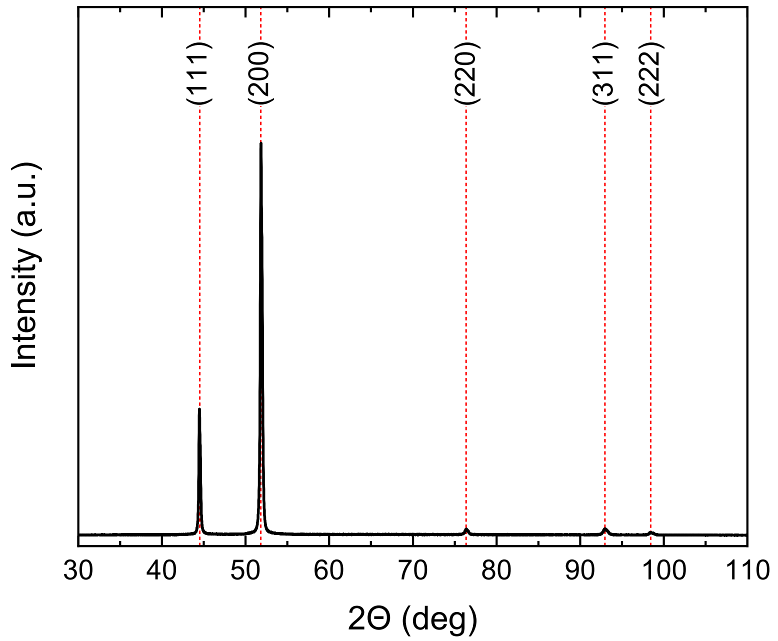

Figure S2 – XRD pattern of polycrystalline Ni foil. The reference diffraction pattern of the Ni metal cubic phase (JCPDS #04-0850) is shown as red dotted vertical lines.

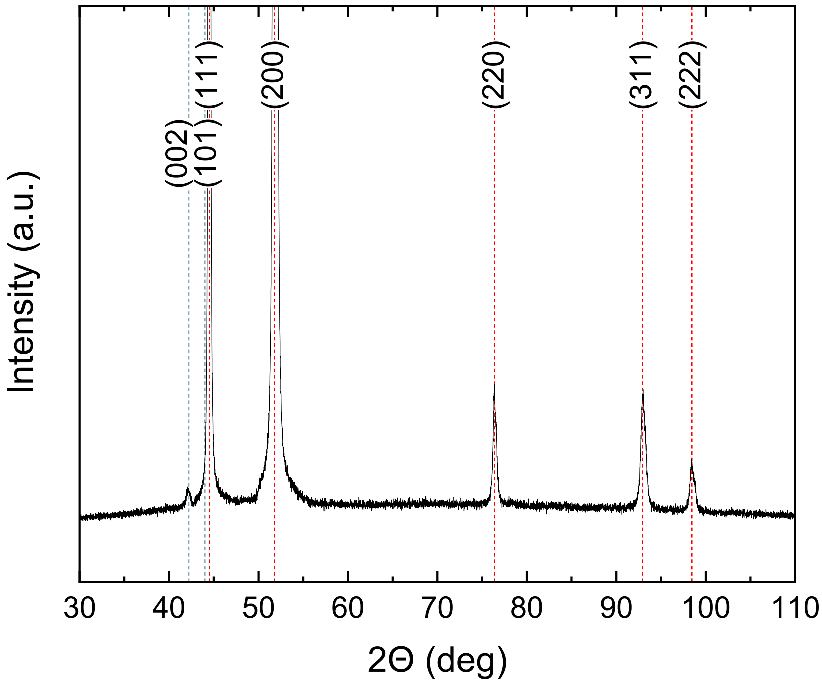

Figure S3– XRD pattern of polycrystalline Ni foil coated with 20 nm of sputter deposited Ru. The reference diffraction pattern of the Ni metal cubic phase (JCPDS #04-0850) and hexagonal close-packed Ru metal *(JCPDS #06-663)* are shown as red and blue dotted vertical lines respectively.

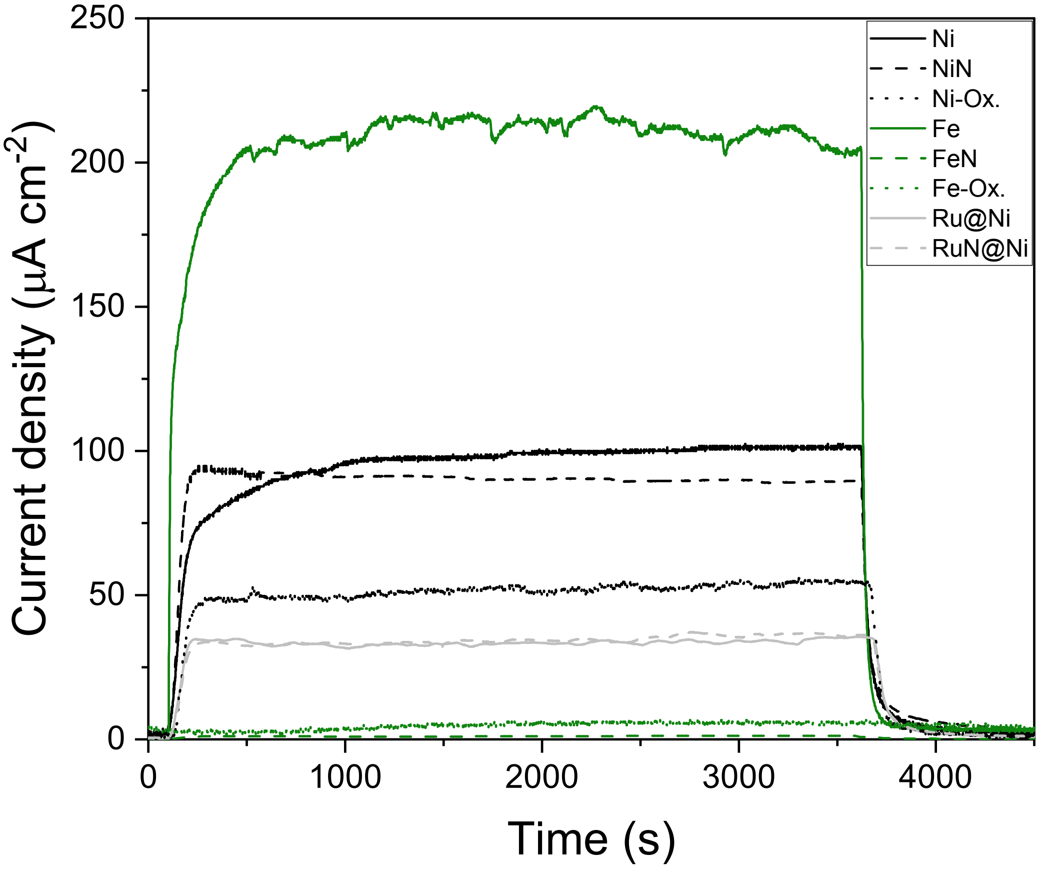

Figure S4 – Measured electrochemical atomic hydrogen permeation rate through different specimens at ambient conditions as a function of time. The y-axis represents the measured current resulting from the oxidation of permeating atomic hydrogen to a proton and an electron at the exit side of the sample; thus this current corresponds to the atomic hydrogen permeation flux. After 100 seconds, the specimen was cathodically polarised in 0.1 M KOH solution at 5 mA cm^{-2}, resulting in the increase in measured oxidation current. The cathodic current was interrupted after 3600 seconds, resulting in the consequent decay of permeated hydrogen. The measured hydrogen permeation transient shows the characteristic s-shaped profile. Further details on the electrochemical hydrogen permeation experiments are available in the experimental section of the manuscript. The analysed specimens are: 12.5 µm nickel foil (black), 25 µm Fe foil (green), and 12.5 µm nickel foil coated with 20 nm Ru (grey). Different line styles indicate the condition of the surface of the specimen: oxidised in air (dotted line), metallic surface (solid line), or with a surface nitride top-layer (dashed black). The clean metallic Ni and Fe surfaces (black and green solid lines) were obtained through a Ar/H_2 plasma treatment described in the experimental session of the manuscript (sample preparation subsection). A protective 20 nm Pd layer was sputter deposited (only for hydrogen permeation measurements) on the hydrogen exit side of all the electrodes after either the oxidation, cleaning or nitriding step. The adoption of a protective Pd layer is common practice in electrochemical hydrogen permeation measurements using a Devanathan-Stachurski cell.[5]

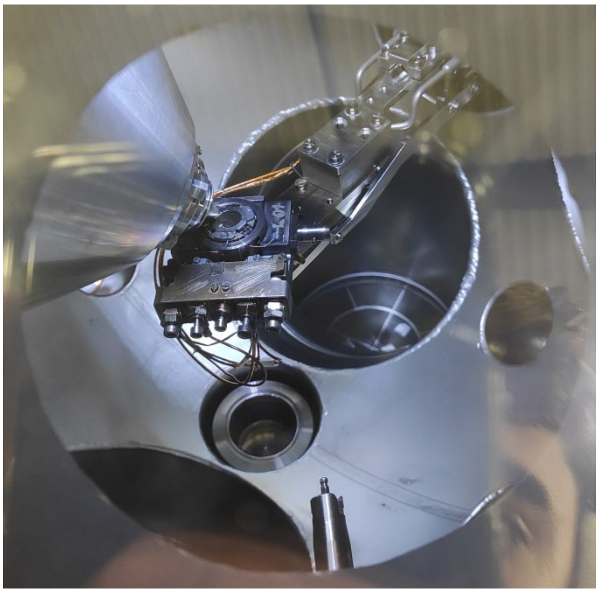

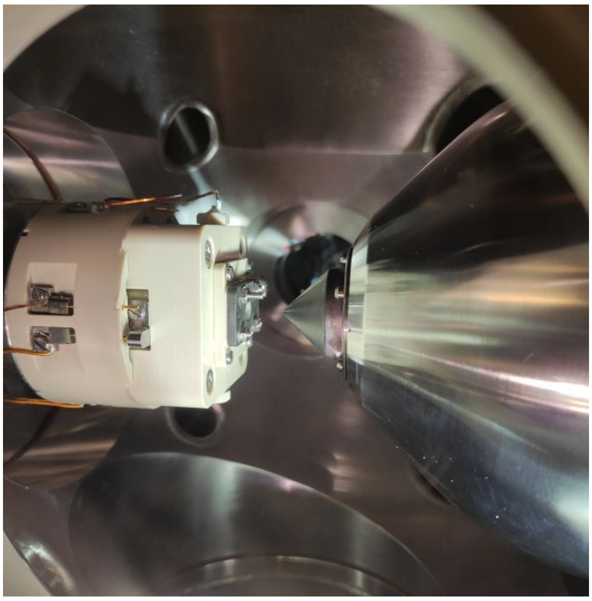

Figure S5 – On the left, a picture of the sample holder for back laser heating experiments in the analysis chamber of the *BL24-CIRCE* beamline (ALBA, Barcelona). On the right, a picture of the *in situ* electrochemical cell within the analysis chamber of the *ISISS* beamline (BESSY II, Berlin).

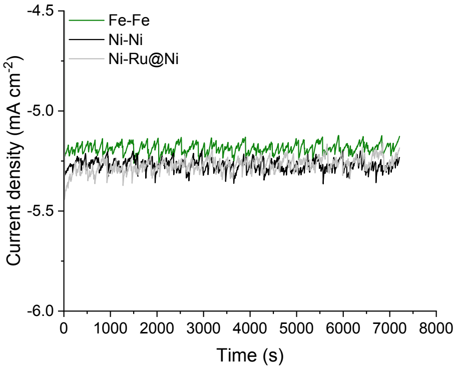

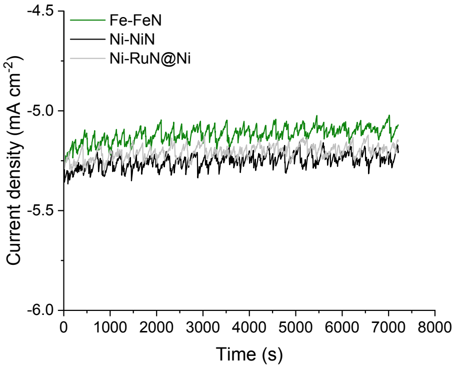

Figure S6 – Electroanalytical measurements from the six types of electrodes tested in this study. All the electrochemical tests were carried out in potentiostatic condition (-1.5 V vs SHE) in a flowing 0.1 M KOH electrolyte solution, using a Pt counter electrode and a saturated Ag/AgCl reference electrode. The material at the electrode-electrolyte interface (either Ni or Fe) is specified in the legend in the left side of the dash, while the material at the electrode-gas interface (Fe, Ni, Ru or their corresponding nitrided surfaces) is indicated on the right side of the dash (so e.g. Ni-RuN@Ni indicates a Ni electrode-electrolyte interface and a RuN@Ni electrode-gas interface..

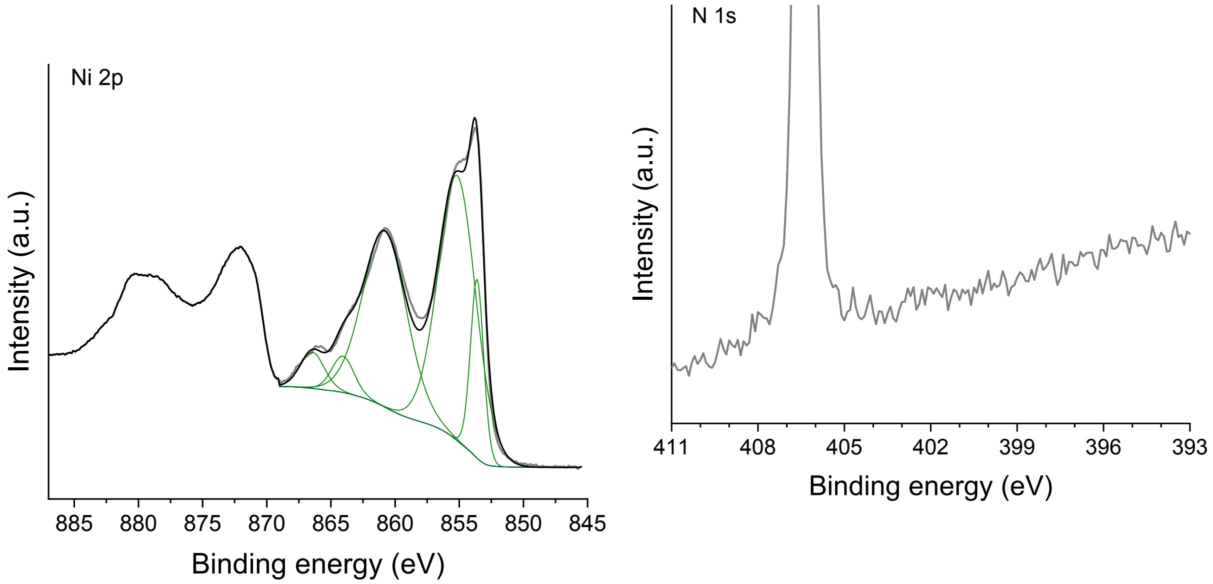

Figure S7 – Ni 2p (left) and N 1s (right) XPS spectra of a nickel oxide layer grown onto a clean Ni foil via in situ exposure to O_2 at 200 °C and subsequently exposed to N_2 (1 mbar). Peak fitting (in green) of Ni 2p spectrum shows a fully oxidised (NiO) nickel surface. The N 1s spectrum does not show any reactivity towards N_2 adsorption. The large peak around 406.5 eV is due to the presence of dinitrogen gas.[6] Photoelectron kinetic energy was 200 eV.

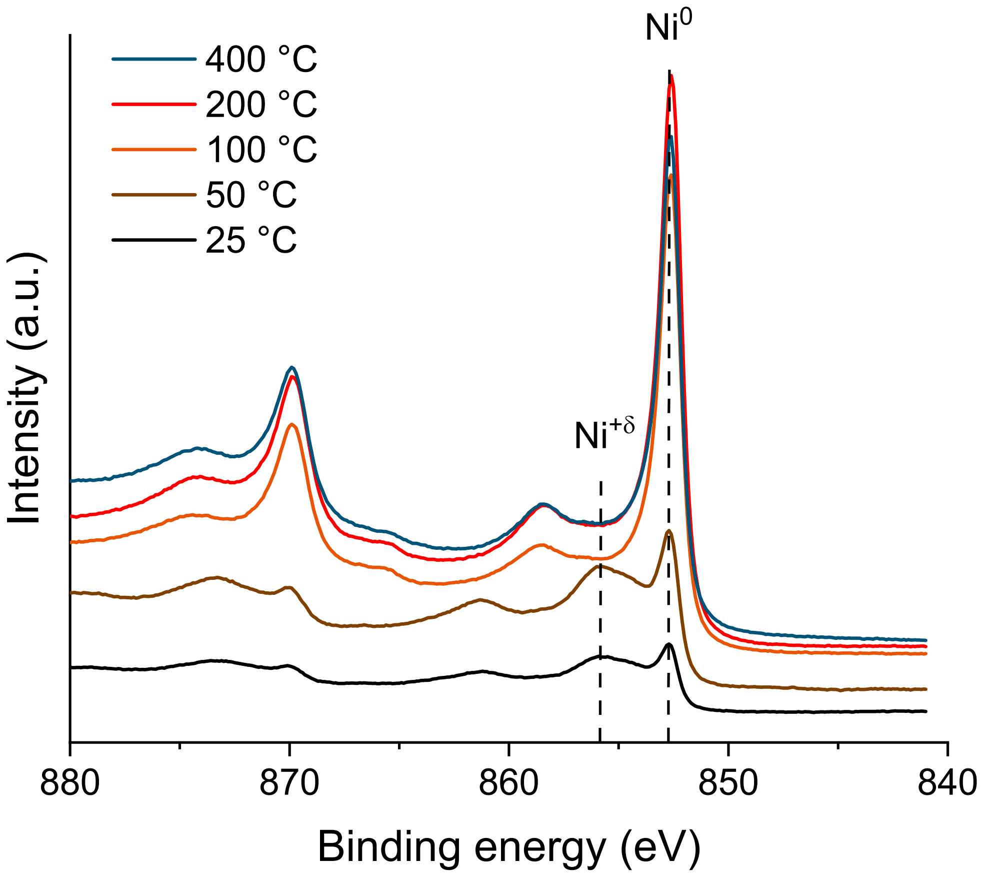

Figure S8 – Ni 2p XPS spectra of the polycrystalline Ni foil in presence of 1 mbar of H_2 heated up from room temperature (25 °C) to 400 °C. The position main metallic and oxide components is indicated by the dashed lines. Reduction of the Ni surface is achieved from 100 °C. Photoelectron kinetic energy was 200 eV.

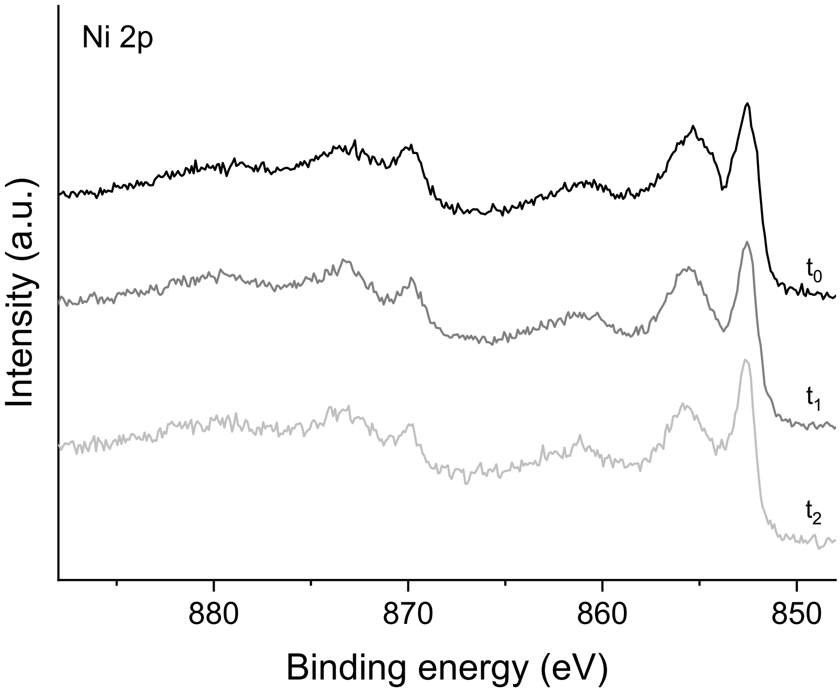

Figure S9 – Stacked Ni 2p XPS spectra of the polycrystalline Ni foil under vacuum and X-ray illumination over time (t_0= 0 min, t_1= 20 min and t_2= 45 min). No noticeable reduction of the Ni surface is observable after 45 min. Photoelectron kinetic energy was 200 eV.

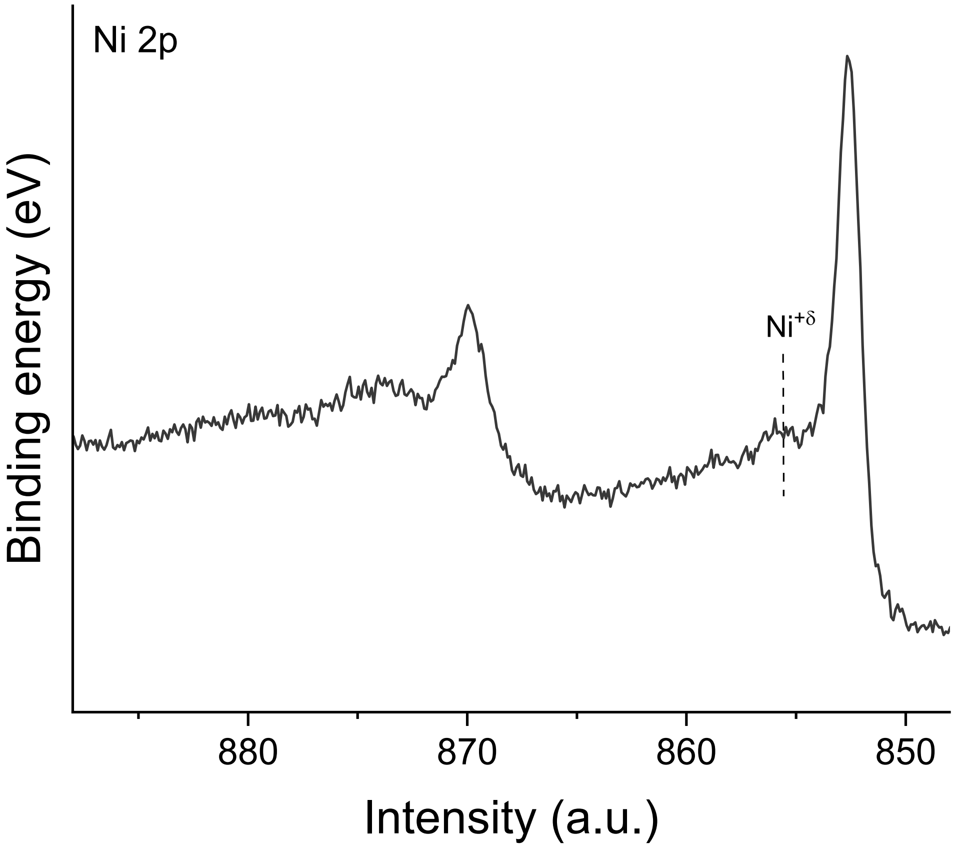

Figure S10 – Ni 2p XPS spectra the polycrystalline Ni foil held for about 15 minutes in OCP under vacuum, after full reduction by permeating atomic hydrogen. Re-oxidation of the electrode surface is noticeable from the distinctive oxide component, here indicated with a dashed line. This is attributed to the presence of residual traces of oxygen and water vapour in the analysis chamber (the pressure of the analysis chamber prior starting the experiment was about 7·10^{-7} mbar). Photoelectron kinetic energy was 200 eV.

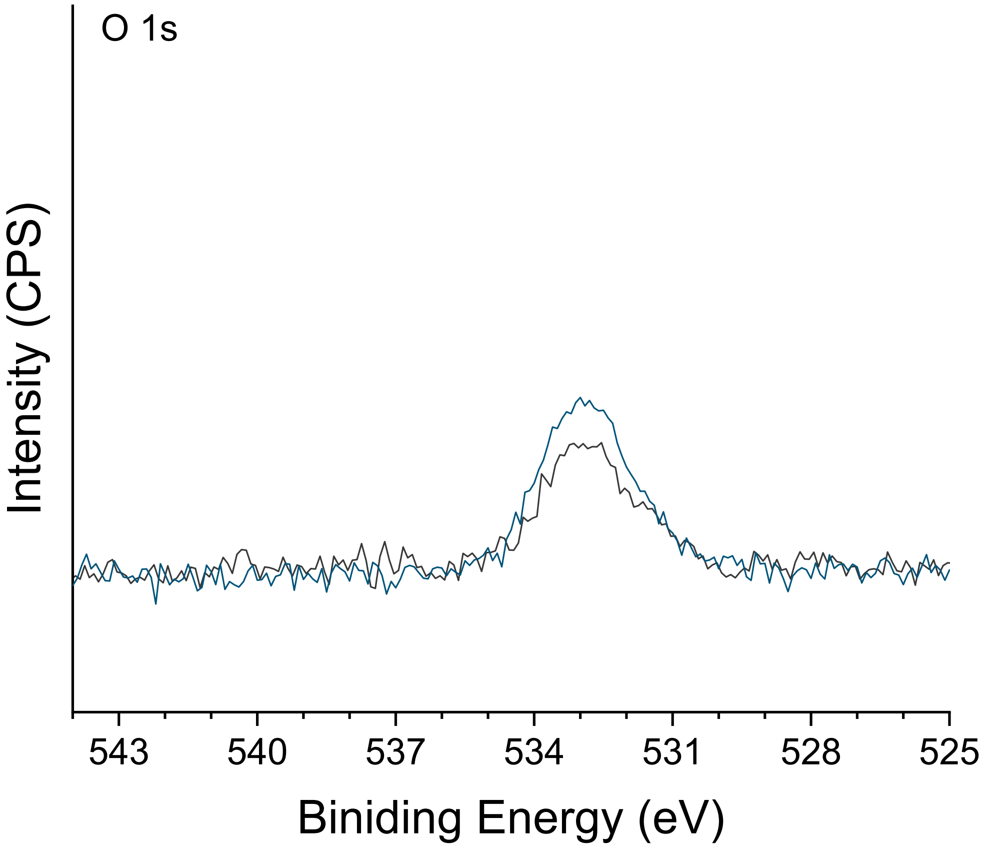

Figure S11 – Comparison of the O 1s spectra of the Ni surface before (black) and after (blue) the exposure to N_2 (0.1 mbar). Photoelectron kinetic energy was 200 eV.

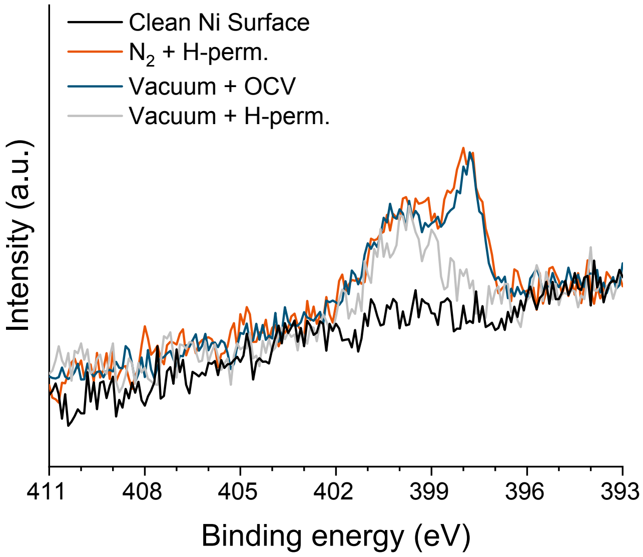

Figure S12 – N 1s XPS spectra of the reduced metallic Ni electrode before the exposure to N_2 showing the absence of initial N on the surface (black), after the exposure to 0.1 mbar of N_2 during electrochemical atomic hydrogen permeation (orange), after subsequent evacuation to UHV and interruption of the hydrogen permeation (blue) and after restoring the electrochemical atomic hydrogen permeation in UHV (grey). Photoelectron kinetic energy was 200 eV.

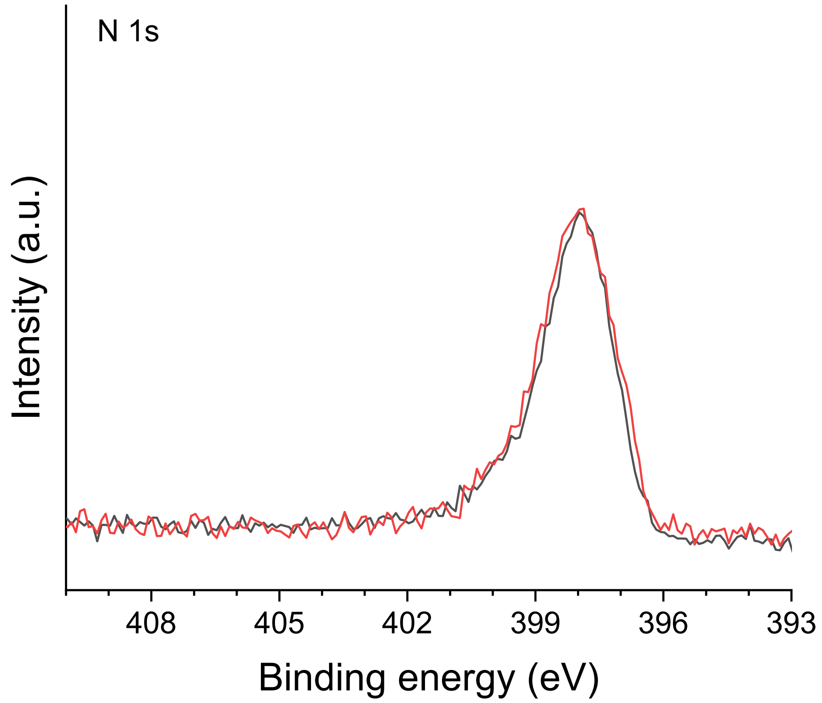

Figure S13 – N 1s XPS spectra of the surface nickel nitride before (black) and after (orange) H_2 exposure (0.1 mbar) at room temperature.

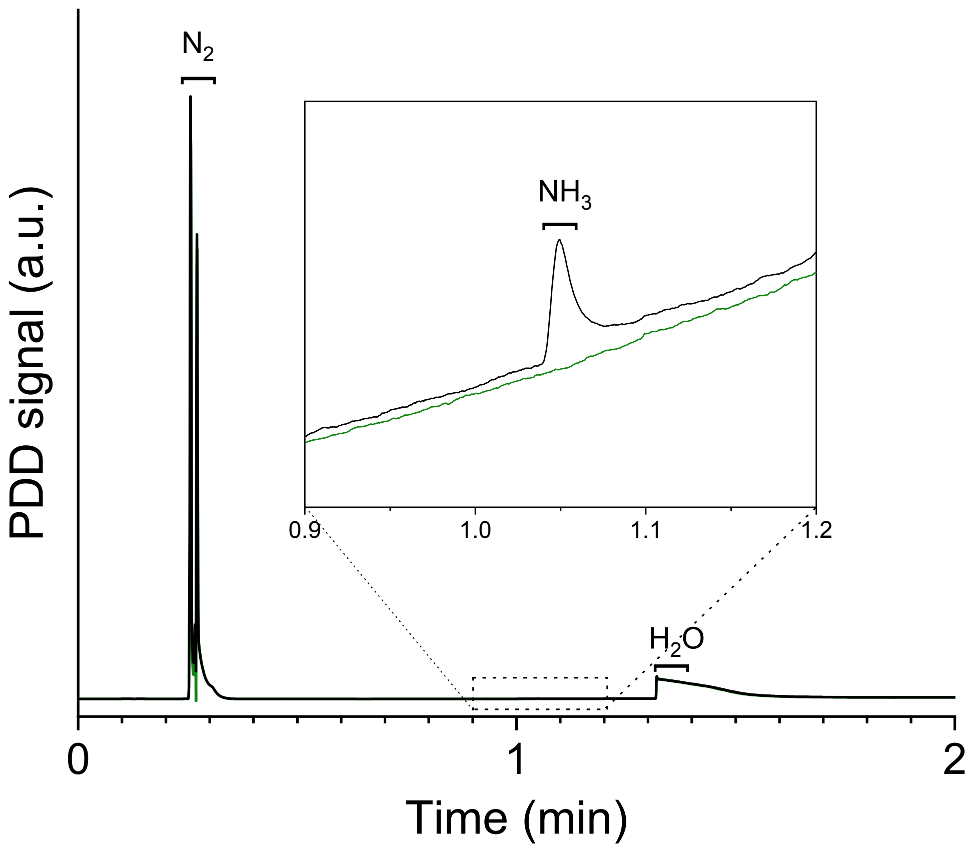

Figure S14 – Full chromatographs of the nitrided Ni surface electrode during NH_3 synthesis experiment. Permanent gases are eluted at 0.25 min, ammonia at 1.05 min, and water at 1.3 min. The enclosure highlights the region of the chromatograph around the ammonia elution time. Ammonia is produced when the electrochemical atomic hydrogen permeation through the electrode is initiated (black line). On the other hand, no NH_3 is detected in OCP (green line), i.e. no H permeation.

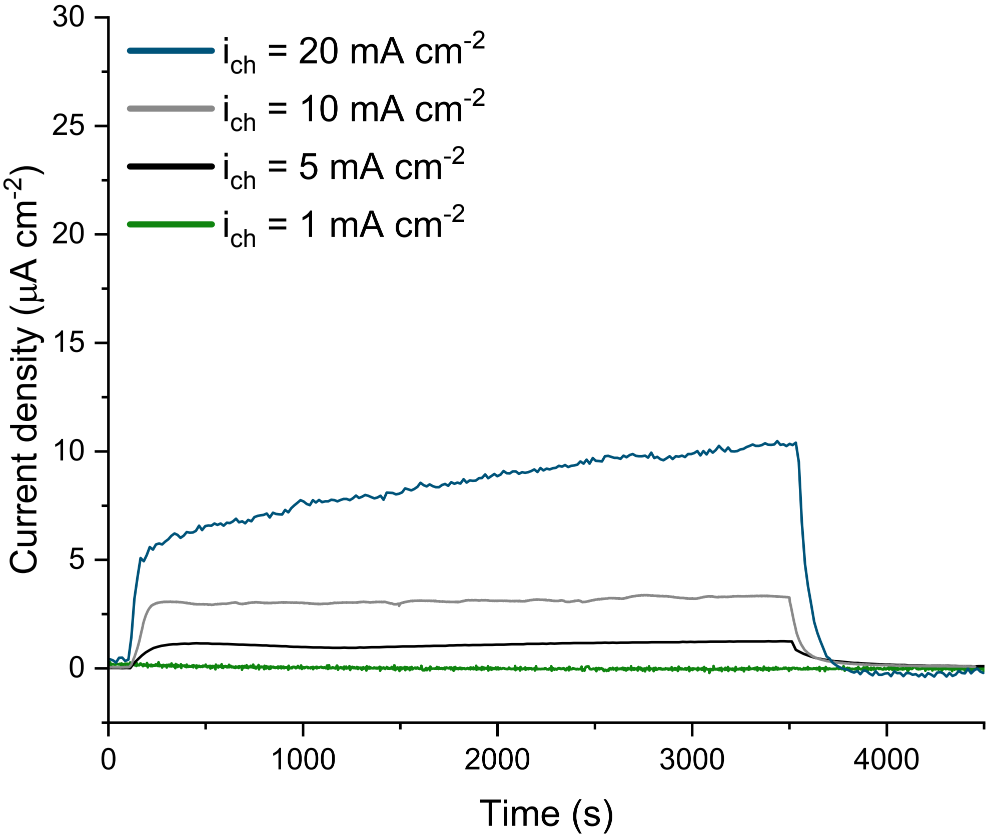

Figure S15 – Measured electrochemical atomic hydrogen permeation rate through 25 µm Fe foil oxidised in air at different charging current densities (1 to 20 mA cm^{-2}). The y-axis represents the measured current resulting from the oxidation of permeating atomic hydrogen to a proton and an electron at the exit side of the sample; thus this current corresponds to the atomic hydrogen permeation flux. After about 100 seconds, the specimen was cathodically polarised in 0.1 M KOH solution, resulting in the increase in measured oxidation current. The cathodic current was interrupted after 3600 seconds, resulting in the consequent decay of permeated hydrogen. The measured hydrogen permeation transient shows the characteristic s-shaped profile. A protective 20 nm Pd layer was sputter deposited (only for hydrogen permeation measurements) on the hydrogen exit side of all the electrodes. Further details on the electrochemical hydrogen permeation experiments are available in the experimental section of the manuscript. Slightly higher H permeation are achieved at higher charging current. However, because of the presence of an oxide surface layer, even at 20 mA cm^{-2} charging current density, the H permeation remain still substantially low (< 10 µA cm^{-2}) and more than 20 times lower than a reduced Fe electrode (Figure S4).

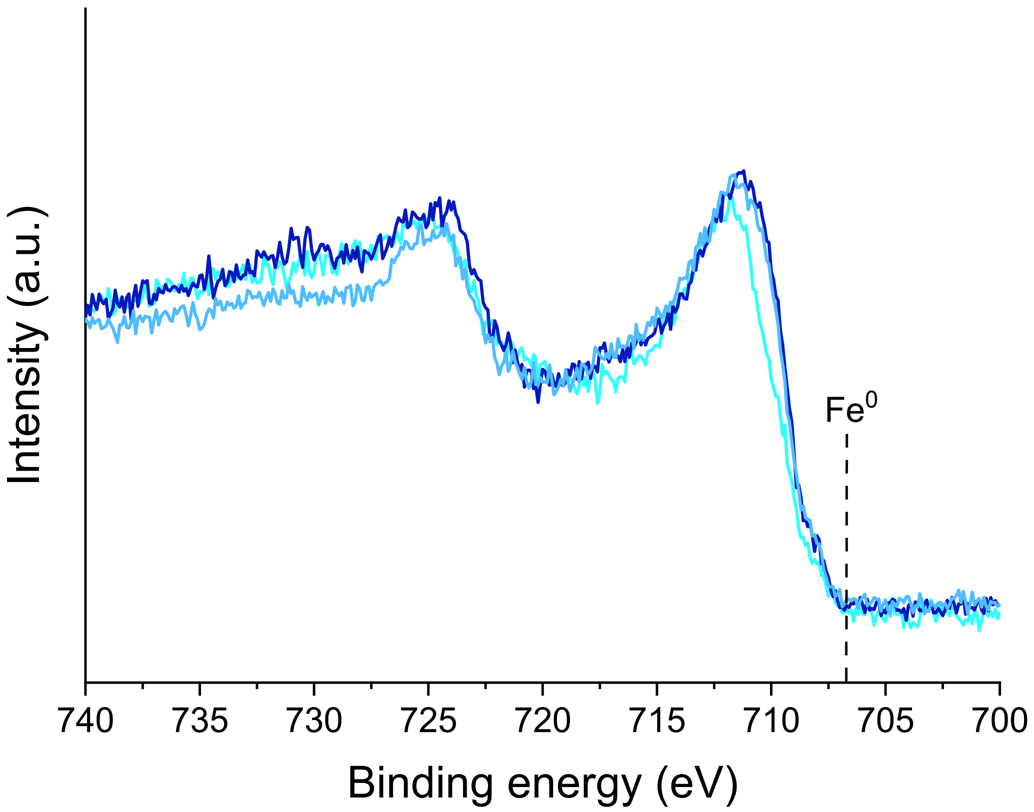

Figure S16 – Fe 2p XPS spectra as received and after about one and two hours of electrochemical hydrogen loading under vacuum (lighter to darker colour indicates increasing time). Metallic Fe species are not observed. Only a slight shift towards lower binding energy is notable, which can be associated to a limited partial reduction of some Fe^{+3} species to Fe^{+2}. Photoelectron kinetic energy was 200 eV.

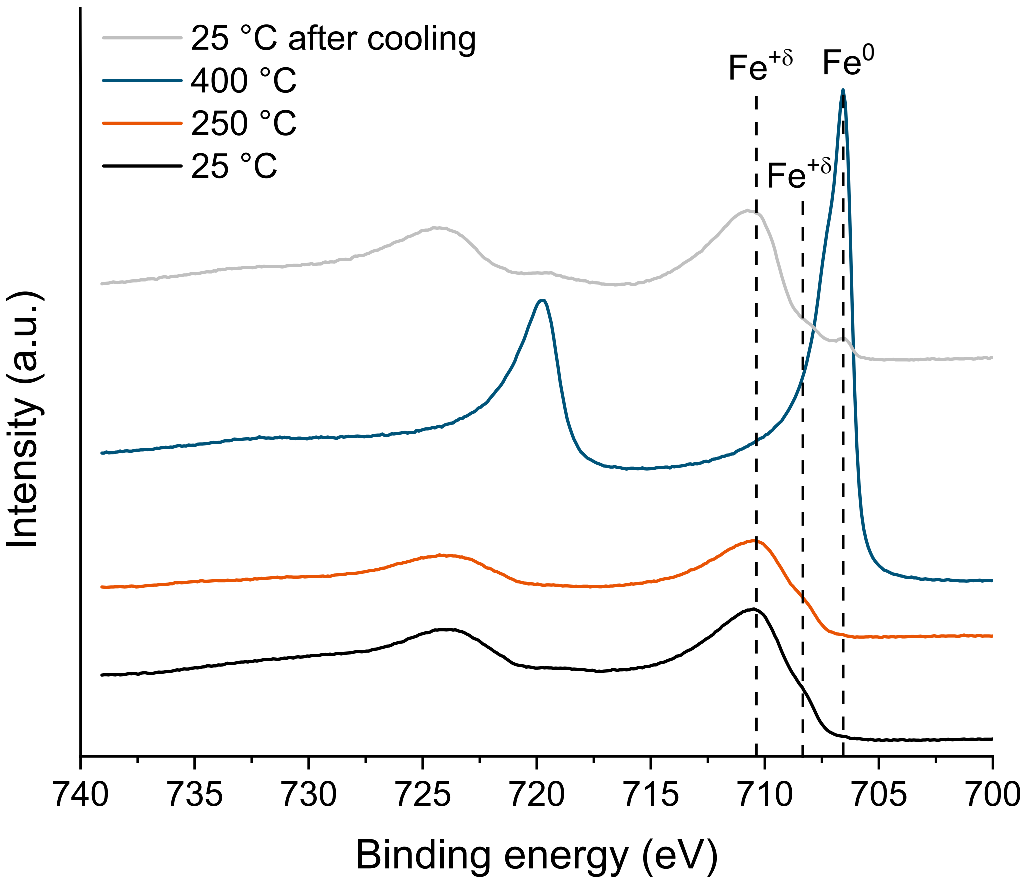

Figure S17 – Fe 2p XPS spectra of the polycrystalline Fe foil in presence of 1 mbar of H_2 heated up from room temperature (25 °C) to 400 °C and then cooled down again to 25 °C (grey line). The position of the main metallic and oxide components is indicated by the dashed lines. Reduction of the Fe surface is achieved for temperatures higher than 250 °C. Photoelectron kinetic energy was 200 eV.

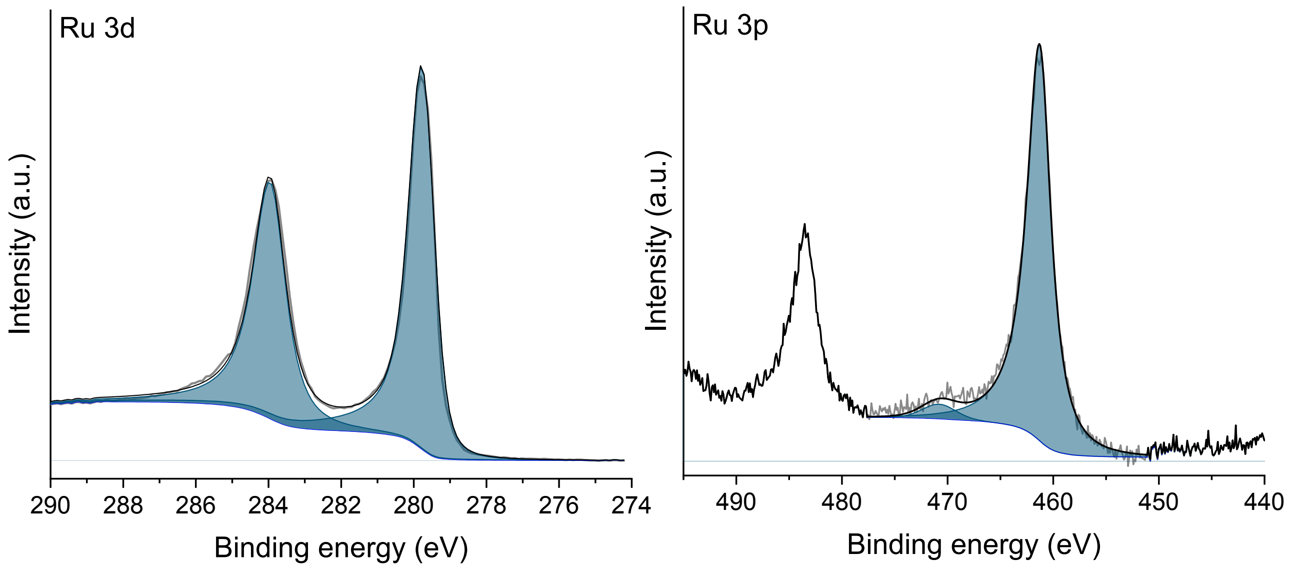

Figure S18 – Ru 3d (left) and Ru 3p (right) XPS spectra showing a metallic ruthenium surface, with peak fitting in agreement with literature.[1] Photoelectron kinetic energy was 300 eV.

Additional XPS spectra

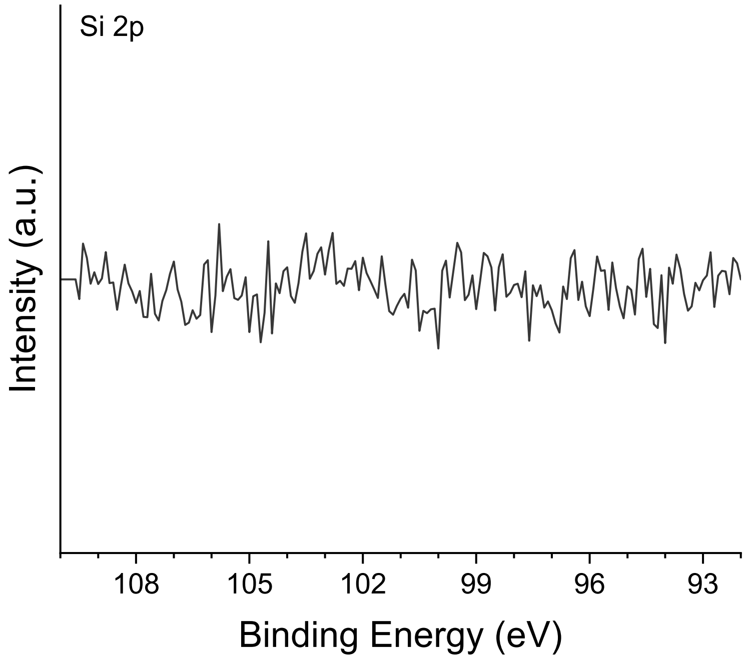

Figure S 19 – Si 2p XPS spectrum of the Ni foil shows the absence of Si in the sample.

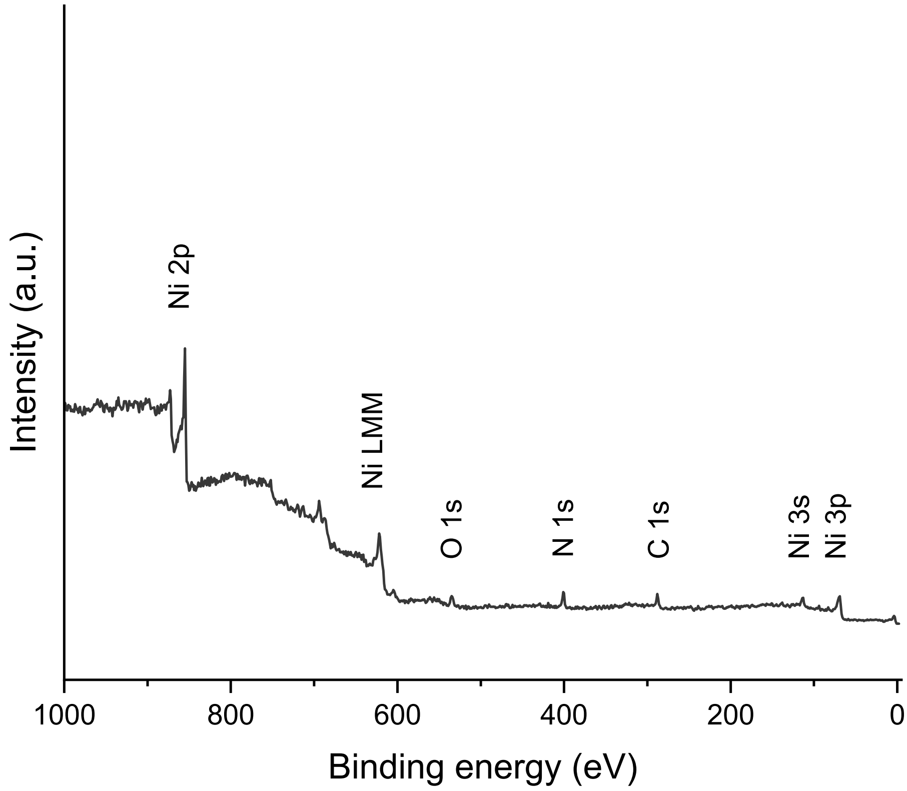

Figure S 20 – XPS survey spectrum of the surface nickel nitride sample, collected with 1467 eV excitation energy. Traces of adventitious C and O are a consequence of unavoidable air exposure.

Table S1. Parameters used for the Ni $2p_{3/2}$ XPS fits using CasaXPS software package: binding energy (BE), line shape, full width at half-maximum (FWHM) and relative peak area. A Shirley background was used as baselines.

Component	BE (eV)	Line shape	FWHM (eV)	Relative area (%)
Ni^0	852.6	LA(1.1,2.2,10)	1.0	100
	856.3	GL(30)	2.5	7.0
	858.7	GL(30)	2.8	16.8
NiO	853.7	GL(30)	1.1	28.4
	855.4	GL(30)	3.0	100
	860.9	GL(30)	3.7	76.9
	864.0	GL(30)	2.0	8.1
	866.4	GL(30)	2.4	8.8
$Ni(OH)_2$	854.9	GL(30)	1.1	16.3
	855.7	GL(30)	2.2	100
	857.7	GL(30)	1.9	6.6
	860.5	GL(30)	1.1	3.1
	861.5	GL(30)	4.4	86.5
	866.5	GL(30)	3.0	8.2

Table S2. Parameters used for the O1s XPS fits using CasaXPS software package: binding energy (BE), line shape and full width at half-maximum (FWHM). A Shirley background was used as baselines.

Component	BE (eV)	Line shape	FWHM (eV)
Oxides	529.3	GL(30)	1.0
Hydroxides	531.1	GL(30)	1.9
H_2O^{ad}	532.9	GL(30)	1.3

Table S3. Parameters used for the N 1s XPS fits using CasaXPS software package: binding energy (BE), line shape, full width at half-maximum (FWHM). A Shirley background was used as baselines.

Conditions	Component	BE (eV)	Line shape	FWHM (eV)
Ni^0, OCP, N_2	N^{atom}	397.8	GL(50)	1.8
Ni^0, -1.5 V vs RHE/H-perm, N_2	N^{atom}	397.8	GL(50)	1.0
	NH_3	400.3	GL(50)	2.4
Ni^0, -1.5 V vs RHE/H-perm, vacuum	NH_3	400.3	GL(50)	2.4
NiN, OCP, vacuum	N^{atom}	397.8	GL(50)	1.0
	N^{vac}	399.1	GL(50)	1.9
NiN, -1.5 V vs RHE/H-perm, vacuum	N^{atom}	397.8	GL(50)	1.0
	N^{vac}/NH_x	399.1	GL(50)	1.9
	NH_3	400.3	GL(50)	2.4
$Fe^{+2/+3}$, OCP, N_2	$N\text{-}Fe^{3+}$	399.1	GL(50)	1.5
	N-O	400.6	GL(50)	1.5
Ru, OCP, N_2	N-Ru	397.3	GL(50)	1.5
	N_2^{ad}	399.0	GL(50)	1.5

Table S4. Parameters used for the Ru 3d XPS fits using CasaXPS software package: binding energy (BE), line shape, full width at half-maximum (FWHM) and relative peak area. A Shirley background was used as baselines.

Component	BE (eV)	Line shape	FWHM (eV)	Relative area (%)
Ru^0 $3d_{5/2}$	279.8	LF(0.8,1.25,500,180)	0.7	100
Ru^0 $3d_{3/2}$	284.0	LF(1.01,1.25,500,50)	1.0	70.0

Table S5. Parameters used for the Ru $3p_{3/2}$ XPS fits using CasaXPS software package: binding energy (BE), line shape, full width at half-maximum (FWHM) and relative peak area. A Shirley background was used as baselines.

Component	BE (eV)	Line shape	FWHM (eV)	Relative area (%)
Ru^0 $3p_{3/2}$	461.2	LF(1,1.2,25,5)	2.6	100
	470.8	GL(30)	3.7	3.5

References

1. Morgan, D. J., Resolving ruthenium: XPS studies of common ruthenium materials. *Surface and Interface Analysis* **2015,** *47* (11), 1072-1079.
2. Divins, N. J.; Angurell, I.; Escudero, C.; Pérez-Dieste, V.; Llorca, J., Influence of the support on surface rearrangements of bimetallic nanoparticles in real catalysts. *Science* **2014,** *346* (6209), 620-623.
3. Miller, A. C.; Powell, C. J.; Gelius, U.; Anderson, C. R., Energy calibration of X-ray photoelectron spectrometers. Part III: Location of the zero point on the binding-energy scale. *Surface and Interface Analysis* **1998,** *26* (8), 606-614.
4. Briggs, D.; Grant, J. T.; Limited, S., *Surface Analysis by Auger and X-ray Photoelectron Spectroscopy*. SurfaceSpectra: 2003.
5. Manolatos, P.; Jerome, M.; Galland, J., Necessity of a palladium coating to ensure hydrogen oxidation during electrochemical permeation measurements on iron. *Electrochimica Acta* **1995,** *40* (7), 867-871.
6. Shah, D.; Bahr, S.; Dietrich, P.; Meyer, M.; Thißen, A.; Linford, M. R., Nitrogen gas (N2), by near-ambient pressure XPS. *Surface Science Spectra* **2019,** *26* (1), 014023.
